# Sclerotioloids A–C: Three New Alkaloids from the Marine-Derived Fungus *Aspergillus sclerotiorum* ST0501

**DOI:** 10.3390/md21040219

**Published:** 2023-03-29

**Authors:** Jun-Qiu Mao, Yao-Yao Zheng, Chang-Yun Wang, Yang Liu, Guang-Shan Yao

**Affiliations:** 1 Key Laboratory of Marine Drugs, The Ministry of Education of China, Institute of Evolution & Marine Biodiversity, School of Medicine and Pharmacy, Ocean University of China, Qingdao 266003, China; 2 Fujian Key Laboratory on Conservation and Sustainable Utilization of Marine Biodiversity, Institute of Oceanography, Minjiang University, Fuzhou 350108, China; 3 Laboratory for Marine Drugs and Bioproducts, Qingdao National Laboratory for Marine Science and Technology, Qingdao 266237, China; 4 Institute for Insect Biotechnology, Justus-Liebig-University of Giessen, 35392 Giessen, Germany; 5 Department of Bioresources of the Fraunhofer Institute for Molecular Biology and Applied Ecology (IME), 35392 Giessen, Germany

**Keywords:** marine-derived fungus, *Aspergillus sclerotiorum*, secondary metabolite, alkaloid, sclerotioloids

## Abstract

Alkaloids, as one of the largest classes of natural products with diverse structures, are an important source of innovative medicines. Filamentous fungi, especially those derived from the marine environment, are one of the major producers of alkaloids. In this study, three new alkaloids, sclerotioloids A–C (**1**–**3**), along with six known analogs (**4**–**9**), were obtained under the guidance of the MS/MS-based molecular networking from the marine-derived fungus, *Aspergillus sclerotiorum* ST0501, collected from the South China Sea. Their chemical structures were elucidated by comprehensive analysis of the spectroscopic data, including 1D and 2D NMR and HRESIMS. Additionally, the configuration of compound **2** was unambiguously determined by X-ray single crystal diffraction, and that of compound **3** was determined by the TDDFT-ECD approach. Sclerotioloid A (**1**) represents the first example of 2,5-diketopiperazine alkaloid with a rare terminal alkyne. Sclerotioloid B (**2**) showed the inhibition of NO production induced by lipopolysaccharide (LPS), with an inhibition rate of 28.92% higher than that of dexamethasone (25.87%). These results expanded the library of fungal-derived alkaloids and further prove the potential of marine fungi in the generation of alkaloids with new scaffolds.

## 1. Introduction

In the past few years, natural products isolated from marine-derived fungi have aroused great interest due to their unique structures, interesting the pharmacological and biological properties [1]. Among the marine-derived fungi, *Aspergillus* is the largest source of fungal natural products. The growth of marine-derived fungi research rose from 2015 to 2019, with natural products from marine-derived fungi sources already accounting for nearly half (47%) of the total number of new marine natural products reported by 2019 [2]. Additionally, a large part of the compounds isolated from *Aspergillus* show anti-bacterial, anti-cancer, anti-inflammatory, anti-tuberculosis, and cytotoxic activities [3]. Metabolites of marine-derived fungi, such as polyketides, alkaloids, terpenes, lactones and peptides, are rich sources of biologically active natural products [4]. It was reported that alkaloids represent about 18% of all the marine natural products in 2019 [2]. Recent studies of marine fungal metabolites looking for bioactive compounds suggest that they have the potential to become a source of new drugs [5,6]. For example, plinabulin is a 2,5-diketopiperazine alkaloid isolated from the marine-derived fungus *Aspergillus* sp., which was first used in clinical trials for the treatment of non-small-cell lung cancer (NSCLC) [7]. Ecteinascidin-743 (ET743) is a novel anti-tumor drug on the market [8]. *Aspergillus sclerotiorum* have potential to biosynthesize alkaloids, though alkaloids are the most bioactive metabolites that possess marked antimicrobial and cytotoxic activities [9]. Sclerotiamides C and F, which are isolated from the *A. sclerotiorum* LZDX-33-4, a fungus derived from a gorgonian coral (LZDX-33), showed a significant inhibitory effect against a panel of tumor cell lines with IC_50_ values ranging from 1.6 to 7.9 μM [10]. A new cytotoxic indole-3-ethenamide isolated from *A*. *sclerotium* PT06-1 showed moderate (3.0 mM) and weak (27 mM) cytotoxicity toward A-549 and HL-60 cells, respectively [11].

During our ongoing research for new bioactive secondary metabolites from marine-derived fungi in the South China Sea [12,13,14,15], plenty of marine-derived alkaloids have been isolated. For example, emestrins L and M were obtained from the marine-derived fungus *A. terreus* RA2905. Emestrin M displayed antibacterial activity against *Pseudomonas aeruginosa* ATCC 27,853, with a minimum inhibitory concentration (MIC) value of 64 µg/mL [16]. Three new indole-diketopiperazine alkaloids, spirotryprostatin G and cyclotryprostatins F and G, were obtained from fungal strain HBU-136, and among them, spirotryprostatin G exhibited cytotoxicity against the HL-60 cell line, with an IC_50_ value of 6.0 μM, while cyclotryprostatins F and G exhibited cytotoxicity against the MCF-7 cell line, with IC_50_ values of 7.6 and 10.8 μM, respectively [17].

The fungal strain *A. sclerotiorum* ST0501 was isolated from the inner part of an unidentified sponge, GDST-2013-05, collected from the South China Sea. In the previous work, three new 2,5-diketopiperazine alkaloids, speramide C, 3,21-*epi*-taichunamide F and 2-*epi*-amoenamide C, were obtained from the same fungus strain. Speramide C represents the first prenylated indole alkaloid with an ethylene oxide ring on the isopentenyl side chain [18]. However, in this paper, we discovered three new alkaloids (**1**–**3**) and six known analogs (**4**–**9**) from ethyl acetate extract of *A*. *sclerotiorum* ST0501 based on the MS/MS molecular network. Herein, we report the isolation, structure elucidation and biological activities of these isolated compounds.

## 2. Results and Discussion

### 2.1. Elucidation of Chemical Structures

**Figure 1:**
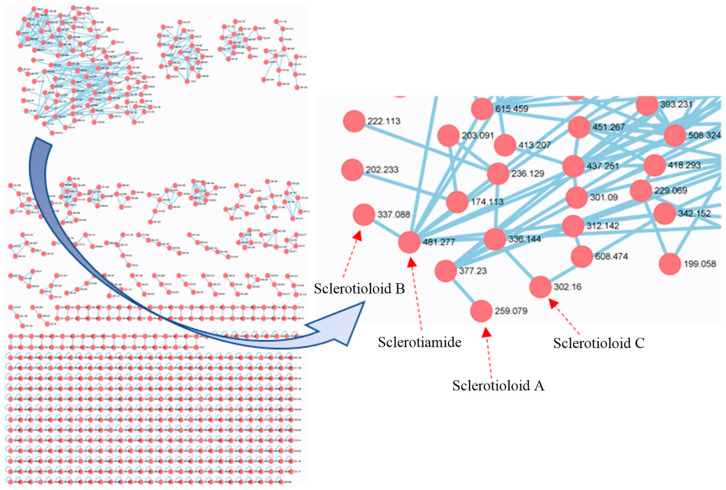
LC-MS/MS-based molecular networking of *Aspergillus sclerotiorum* ST0501.

**Figure 2:**
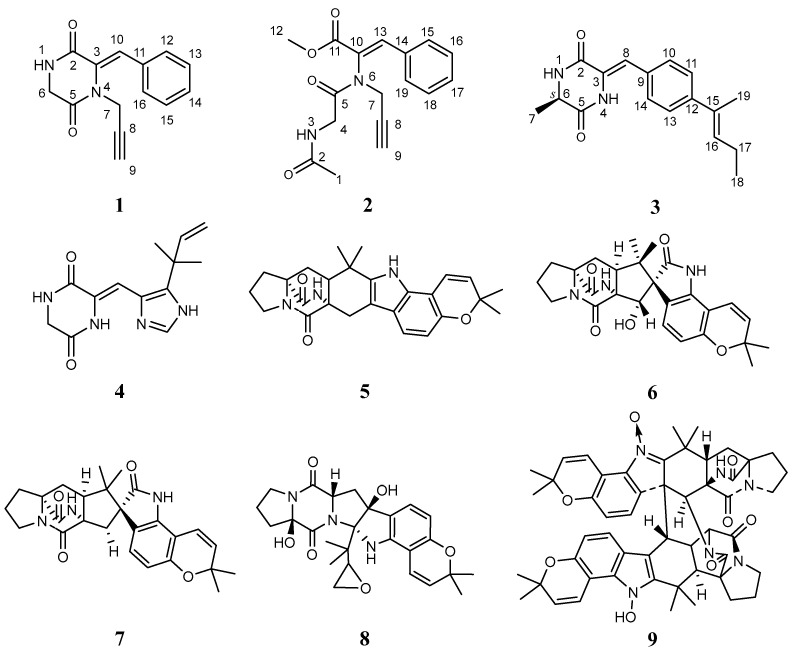
The structure of **1**–**9**.

In order to fully explore the alkaloids of ST0501, molecular networking analysis was performed on the crude ethyl acetate extract (EtOAc) of solid rice and wheat cultures of *A. sclerotiorum* ST0501. The results show that there are signals associated with alkaloids. The relationship between compound **2** and compound **6** is close. Based on the indication of molecular networking (Figure 1), three unreported (**1**–**3**) and six previously described alkaloids (**4**–**9**) (Figure 2) were isolated by using a combination of column chromatography including silica gel, octadecylsilyl, Sephadex LH-20 columns and semi-preparative HPLC. The structures of gartryprostatin C (**4**) [19], stephacidin A (**5**) [20], sclerotiamide (**6**) [21], notoamide B (**7**) [22], speramide C (**8**) [18] and stephacidin B (**9**) [23] were elucidated by comparison of their spectroscopic data with those previously reported in the literature.

**Figure 3:**
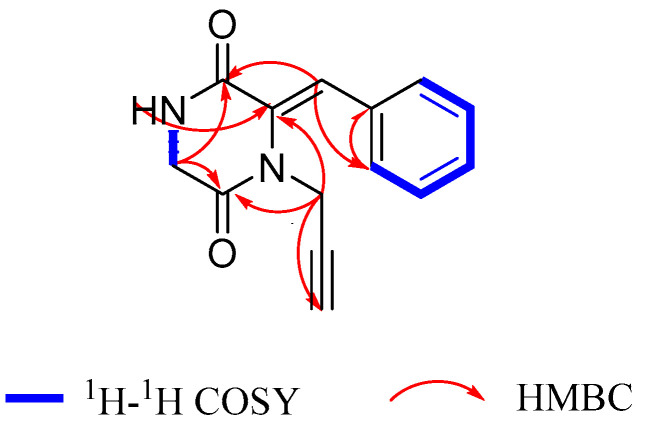
The key ^1^H-^1^H COSY and HMBC correlations of compound **1**.

Sclerotioloid A (**1**) was isolated as a yellow powder. Its molecular formula C_14_H_12_N_2_O_2_ was deduced from its HRESIMS *m*/*z* 263.0794 [M+Na]^+^ (calcd for C_14_H_12_N_2_O_2_Na, 263.0797) and 1D NMR data analysis, suggesting ten degrees of unsaturation. The IR spectrum of **1** featured typical absorption bands for alkyne (2361 cm^−1^) and conjugated ketone (1698 cm^−1^). The ^1^H NMR spectrum of **1** (Table 1) exhibited one amine proton (*δ*_H_ 8.42, s, 1-NH), five aromatic protons (*δ*_H_ 7.35–7.40, m, 1-substituted benzene ring), one olefinic proton (*δ*_H_ 7.10, s, H-10), two methylene protons (*δ*_H_ 4.13 d, *J* = 2.4 Hz, H-7; *δ*_H_ 4.01 d, *J* = 2.3 Hz, H-6) and one acetylenic proton (*δ*_H_ 3.10, t, *J* = 2.4 Hz, H-9). The ^13^C NMR spectrum, in combination with HSQC spectra, revealed the presence of fourteen carbons, including two amide carbonyl (*δ*_C_ 165.7, C-5 and *δ*_C_ 163.4, C-2), six aromatic carbons (*δ*_C_ 133.4, C-11; *δ*_C_ 129.4, C-13 and C-15; *δ*_C_ 128.8, C-14; *δ*_C_ 128.6, C-12 and C-16), two olefinic carbons (*δ*_C_ 129.9, C-3 and *δ*_C_ 120.8, C-10), two alkynyl carbons (*δ*_C_ 78.2, C-8 and *δ*_C_ 74.5, C-9) and two methylene *sp*^3^ (*δ*_C_ 44.5, C-6 and *δ*_C_ 32.9, C-7). The correlations between H-1 and H-6 in the ^1^H-^1^H COSY spectrum and the HMBC correlations between NH-1 and C-3 and between H-6 and C-2/C-5 revealed the existence of a 2,5-diketopiperazine ring. Furthermore, the HMBC correlations between H-10 and C-2/C-3/C-16 indicated that the benzene ring was connected to C-3 of the 2,5-diketopiperazine ring through C-10. Based on the HMBC correlations between H-7 and C-3/C-5/C-9, the alkynyl group was connected to N-4 of the 2,5-diketopiperazine ring (Figure 3). According to the (*Z*)-vinyl proton chemical shifts of H-7 (*δ*_H_ 7.03) in nocazine C [24] and H-7 (*δ*_H_ 6.54) in (3*S*,6*E*)-3-benzyl-6-benzylidenepiperazine-2,5-dione [25], the Δ^3,10^ double bond in **1** was deduced to have a *Z* configuration due to the relative downfield shift of H-10 (*δ*_H_ 7.10), where the (*Z*)-vinyl proton had a larger downfield shift than that of the (*E*)-vinyl proton because of the deshielding effect of the carbonyl in 2,5-diketopiperazine [24,25,26,27]. Therefore, the structure of **1** was elucidated. 

**Figure 4:**
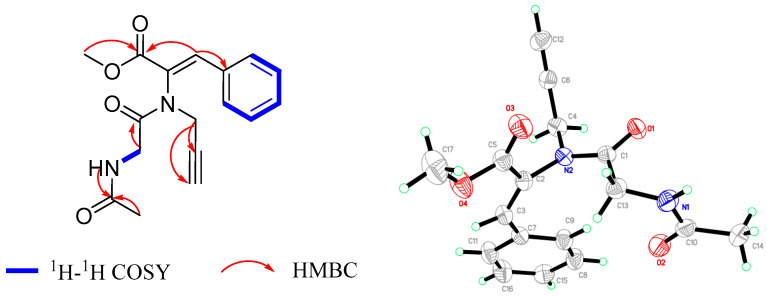
The key ^1^H-^1^H COSY and HMBC correlations of **2** (left) and the ORTEP diagram of **2** (right).

Sclerotioloid B (**2**) was also isolated as a yellow powder. The HRESIMS spectrum of **2** indicates a molecular formula of C_17_H_18_N_2_O_4_ based on the prominent peak [M+Na]^+^ at *m*/*z* 337.1160 (calcd for C_17_H_18_N_2_O_4_Na, 337.1164), as well as 1D NMR data analysis, requiring ten degrees of unsaturation. The IR spectrum of **2** featured typical absorption bands for alkyne (2361 cm^−1^) and conjugated ketone (1557 cm^−1^). The ^1^H NMR and ^13^C NMR spectroscopic data of **2** (Table 2) showed high similarity with **1**, except for the appearance of one methoxyl protons resonating at *δ*_H_ 3.81 (H-12) and one methyl protons resonating at *δ*_H_ 1.77 (H-1), as well as the additional carbonyl carbon resonating at *δ*_C_ 169.0 (C-2). The existence of an acetamide group was suggested by the HMBC correlations between NH-3 and C-2, between H-1 and C-2 and between H-4 and C-5, as well as the COSY correlation between NH-3 and H-4. The additional HMBC correlations between H-12 and C-11 and between H-13 and C-11/C-15 proved that the benzene ring is connected to the methyl acrylate portion at C-13 (Figure 4). Finally, a single crystal of **2** was obtained after one week of slow crystallization in 95% MeOH (H_2_O) at 4 °C by optimizing the conditions. Therefore, the structure of **2** was confirmed by X-ray crystal diffraction analysis, and this further proved the *Z* configuration of the Δ^10,13^ double bond.

**Figure 5:**
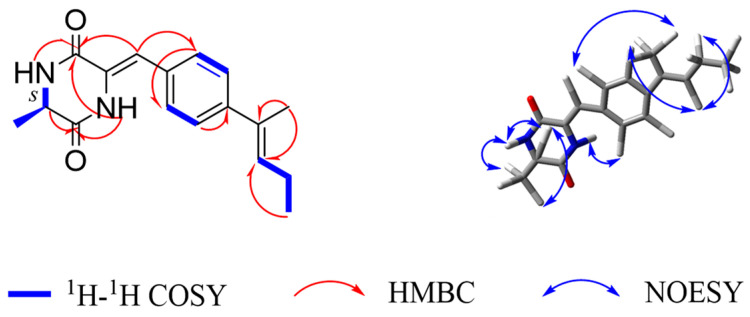
Key ^1^H-^1^H COSY, HMBC and NOESY correlations of **3**.

**Figure 6:**
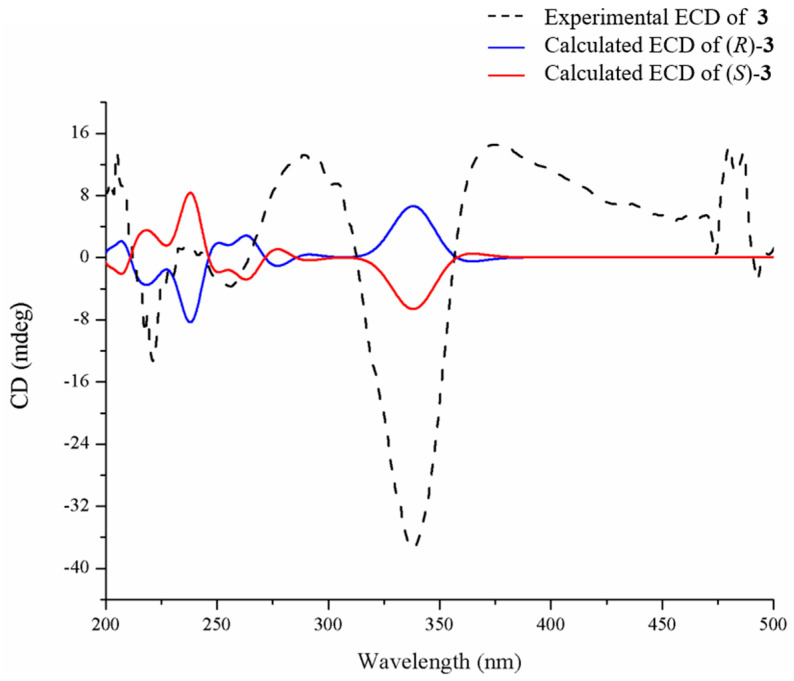
Experimental and calculated ECD spectra of **3**.

Sclerotioloid C (**3**) was isolated as a white powder. Its molecular formula, C_17_H_20_N_2_O_2_, is based on its HRESIMS *m*/*z* 323.1370 [M+K]^+^ (calcd for C_17_H_20_N_2_O_2_K, 323.1162) and 1D NMR data analysis, suggesting nine degrees of unsaturation. The IR spectrum of **3** featured typical absorption bands for olefin (2923 cm^−1^) and conjugated ketone (1512 cm^−1^). The ^1^H NMR spectra of **3** (Table 3) displayed two amine protons (*δ*_H_ 9.79, s, 4-NH; 8.35-8.37, m, 1-NH), four aromatic protons (*δ*_H_ 7.44, d, *J* = 8.8 Hz, H-10 and H-14; *δ*_H_ 6.95, d, *J* = 8.8 Hz, H-11 and H-13), two olefinic protons (*δ*_H_ 6.64, s, H-8; *δ*_H_ 5.43, tt, *J* = 6.7, 1.4 Hz, H-16), one methylene (*δ*_H_ 4.55, d, *J* = 6.7 Hz, H-17), one methine (*δ*_H_ 4.11, d, *J* = 6.8 Hz, H-6), and the protons of three methyl groups (*δ*_H_ 1.74, s, H-19; *δ*_H_ 1.71, d, *J* = 1.3 Hz, H-18; *δ*_H_ 1.33, d, *J* = 7.0 Hz, H-7). ^13^C NMR and HSQC data revealed the presence of seventeen carbons, including two amide carbonyl (*δ*_C_ 167.6, C-2 and *δ*_C_ 160.7, C-5), ten *sp*^2^ resonated between *δ*_C_ 158.2 and *δ*_C_ 114.3, one methylene *sp*^3^ (*δ*_C_ 64.3, C-17), one methine *sp*^3^ (*δ*_C_ 50.3, C-6) and three methyl (*δ*_C_ 25.4, C-19; *δ*_C_ 19.2, C-7; *δ*_C_ 18.0, C-18) carbons. The COSY correlations between NH-1 and H-6 and between H-6 and H-7 and the HMBC correlations between NH-4 and C-2/C-5/C-6, between H-6 and C-2/C-5/C-7 and between H-7 and C-2/C-6 revealed the existence of the 2,5-diketopiperazine ring. The COSY correlations between H-6 and H-7 and the HMBC correlations between H-7 and C-6 revealed that the methyl group is connected to C-6 of the 2,5-diketopiperazine ring. The COSY correlations between H-13 and H-14 and the HMBC correlations between H-12 and C-13/C-14 and between H-14 and C-10 revealed the existence of 1,4-disubstituted benzene ring. The HMBC correlations between H-8 and C-2/C-10 and between H-10 and C-3 indicate that the 2,5-diketopiperazine ring is connected to C-9 of the benzene ring through C-8. The COSY correlations between H-16 and H-17 and between H-and to H-18 and the HMBC correlations between H-17 and C-12/C-16, between H-18 and C-15/C-16 and between H-19 and C-15/C-16, indicate that the *cis*-2-pentene is connected to C-12 of the benzene ring (Figure 5). Therefore, the planar structure of **3** was elucidated as a new diketopiperazine derivative. The configuration of the double bond in **3** was established by the analysis of its NOESY spectrum (Figure 5). The NOESY spectrum did not show any correlation between H-16 and H-19, confirming the *E* configuration of the double bond between C-15 and C-16. In addition, there was no NOESY correlation between H-8 and 4-NH, suggesting *Z* configuration of the double bond between C-3 and C-8. The absolute configuration of C-6 in **3** was determined by the TDDFT-ECD approach. The experimental ECD spectrum of **3** matched well with the calculated ECD spectrum of *S*-**3** (Figure 6) and further supported its absolute configuration.

### 2.2. Bioassays of Compounds

#### 2.2.1. Cytotoxicity Assay

Compound **3** was screened for inhibitory activity on 20 human tumor cell lines at a concentration of 20 μM. However, it showed no observable activities against the abovementioned tumor cell lines (Table S1).

#### 2.2.2. Anti-Microbial Activity Assay

Compounds **1**–**9** were tested for the anti-microbial activity against 20 pathogenic bacteria, including *Enterobacter cloacae*, *E. hormaechei*, *Aeromonas salmonicida*, *Escherichia coli*, Pseudomonas fulva, Photobacterium halotolerans, P. aeruginosa, Staphylococcus aureus, *Bacillus subtilis*, *Vibrio Parahemolyticus* HUB183, *V. alginolyticus* HUB184, *V. anguillarum* HUB185, *Staphylococcus aureus* ATCC 43300, *Enterococcus faecalis* ATCC 51299, *E. faecium* ATCC 35667, S. aureus ATCC 33591, S. aureus ATCC 29213, S. aureus ATCC 25923, *V. vulnificus* MCCC E1758, *V. campbellii* MCCC E333 and two pathogenic fungi, *Candida albicans* ATCC 24,433 and C. parapsilosis ATCC 22019. The initial screening concentration was 50 μg/mL, and none of them showed observable activities against abovementioned pathogens.

#### 2.2.3. Anti-Oxidant Activity Assays

To test the anti-oxidant activity, 1,1-diphenyl-2-picrylhydrazyl (DPPH) assay was used. The initial screening concentration was 50 μg/mL, but none of them showed anti-oxidant activity.

#### 2.2.4. Anti-Inflammatory Activity Assays

Compounds **1**–**3** were screened for NO production inhibitory activity. Compound **2** showed inhibition of NO production induced by lipopolysaccharide (LPS), with an inhibition rate of 28.92 ± 3.49% (Table S2). Compound **2** has a higher inhibition rate than dexamethasone does, which makes it promising as a good anti-inflammatory drug.

Compared with other alkaloid compounds, the activity of compounds **1**–**3** is not good, and other activity models need to be further screened.

## 3. Materials and Methods

### 3.1. General Experimental Procedure

Optical rotations were measured by using a JASCO P-1020 digital polarimeter (589 nm, 20 °C) (JASCO Ltd., Tokyo, Japan). UV spectra were recorded using an HITACHI UH 5300 UV spectrophotometer (Hitachi, Tokyo, Japan). IR spectra were acquired using a Nicolet-Nexus-470 spectrometer in KBr discs (400–4000 cm^−1^) (PerkinElmer Ltd., Boston, MA, USA). NMR spectra were recorded using a JEOL JEM-ECP NMR spectrometer (JEOL Ltd., Tokyo, Japan) with 500 MHz for ^1^H NMR and 125 MHz for ^13^C NMR and using TMS as an internal standard. HRESIMS were analyzed using a Thermo MAT95XP high resolution mass spectrometer (Thermo Fisher Scientific, Bremen, Germany), and ESIMS spectra were analyzed using a Thermo DSQ EImass spectrometer (Thermo Fisher Scientific, Bremen, Germany). Single-crystal X-ray crystallographic analysis was performed using a Bruker D8 venture X-ray single crystal diffractometer (Bruker, Karlsruhe, Germany). HPLC analysis was performed using an HITACHI L-2000 HPLC system coupled with a L-2455 photodiode array detector and using a semi-prepared C18 column (Kromasil 250 mm × 10 mm, 5 µm). For HPLC analysis, the mobile phase consisted of methanol (A) and water (B) with a flowrate of 2 mL/min with 10 µL injection volume (10 mg/mL) and monitored at UV length of 190–400 nm. The elution gradient was as follows: 0–40 min, 40–100% A; 40–45 min, 100% A; 45–50 min, 100–40% A; 50–55 min, 40% A. Sephadex LH-20 (Amersham Biosciences Co., Buckinghamshire, UK) and octadecylsilyl silica gel (Unicorn; 45–60 µm) were used for column chromatography (CC). Precoated silica gel plates (Yan Tai Zi Fu Chemical Group Co.; G60, F-254) were used for thin-layer chromatography analysis. 

### 3.2. Fungal Material

The fungal strain, *A. sclerotiorum* ST0501, was isolated from the inner part of an unidentified sponge, GDST-2013-05, collected from the South China Sea (Guangdong, China) in May 2013. The fungal identification was performed by analysis of its morphological characteristics and ITS region of the rDNA [19]. The sequence data were submitted to Genbank with accession number MT534582. The strain was deposited in a −80 °C refrigerator in the laboratory.

### 3.3. Fermentation, Extraction and Isolation

The fungal strain was cultivated on a solid rice and wheat medium (3.6 g of natural sea salt from Yangkou saltern, China; 80 g of rice; 20 g wheat; 100 mL of H_2_O in 1000-mL Erlenmeyer flask) for 30 d at room temperature. Total fermentation of 45 flasks was extracted repeatedly with equal volume of ethyl acetate (EtOAc) two times, and the organic solvent was evaporated to dryness under vacuum to give a crude extract of 24.0 g. The crude extract was first fractionated by silica gel column chromatography (CC) using a step gradient elution with EtOAc-petroleum ether (0–100%), then with methanol-EtOAc (0–100%) to provide eleven fractions (Fr.1–Fr.11). Fr.3 was separated into three subfractions (M1–M3) by silica gel CC eluted with a step gradient of petroleum ether-EtOAc (from 8/1 to 1/5, *v*/*v*). M1 was placed in a Sephadex LH-20 column and eluted with dichloromethane-methanol (1/1, *v*/*v*) to generate fourteen subfractions (N1–N14). N14 was purified by HPLC (50% methanol-H_2_O) to yield **1** (1.0 mg) and **2** (6.4 mg). Fr.6 was separated into eight subfractions (O1–O8) by silica gel CC eluting with a step gradient of methanol–dichloromethane (0–100%). O4 was further separated by the silica gel CC eluted with a step gradient of dichloromethane–methanol (from 100/1 to 5/1, *v*/*v*) to produce **3** (8.8 mg). Fr.7 was separated into eighteen subfractions (P1–P18) in an ODS column eluted with 30–100% methanol–H_2_O to give **4** (4.5 mg). P6 was separated by the silica gel CC eluted with dichloromethane–methanol (30/1, *v*/*v*) to produce **9** (4.8 mg). P12 was further separated by the silica gel CC eluted with dichloromethane-methanol (60/1, *v*/*v*) to generate **5** (2.3 mg). P13 was separated by the silica gel CC eluted with dichloromethane-methanol (20/1, *v*/*v*) to produce **8** (1 mg). Fr.8 was separated into five subfractions (Q1–Q5) by the silica gel CC eluted with a step gradient of dichloromethane-methanol (60/1 to 5/1, *v*/*v*) to produce **6** (116.5 mg). Q4 was separated into five subfractions (R1–R5) in the Sephadex LH-20 column and eluted with dichloromethane–methanol (1/1, *v*/*v*). R2 was further purified by HPLC (80% methanol–H_2_O) to produce **7** (1.9 mg). 

### 3.4. LC-MS/MS and Molecular Networking Analysis

LC-MS/MS was performed using a Waters series 2695 HPLC instrument coupled with an amaZon SL ion trap Mass spectrometer (Bruker, Karlsruhe, Germany), with a Xchange C18 column (Acchrom Co., CO, USA) 250 mm × 4.6 mm, 5 μm, 0.5 mL/min). The organic portion was dissolved in MeOH at 10 mg/mL, filtered through a Gracepure C18 SPE cartridge and analyzed by LC–MS/MS. Ten μL aliquot of each sample was injected and eluted with a gradient program of MeOH-H_2_O (0.1% formic acid) (0–20 min 10–100%, 21–25 min 100%; 1.0 mL/min). Mass spectra were obtained in positive ESI mode and with an automated fully dependent MS/MS scan from 100 to 1000 Da. MS/MS data were converted digitally to mzXML files using Filezilla software. The molecular networking was performed using the GNPS data analysis workflow using the spectral clustering algorithm [28]. The spectral networks were imported into Cytoscape 3.9.1 and visualized using the force-directed layout.

### 3.5. Spectroscopic and Spectrometric Data

Sclerotioloid A (1): yellow powder; UV (MeOH) λ_max_ (log ε) 221 (0.13), 288 (0.16) nm; HRESIMS (*m*/*z* 263.0794 [M+ Na]^+^, calcd for 263.0797); IR (KBr) *ν*_max_ 3749, 2926, 2361, 1557, 1398, 1053 cm^−1^; ^1^H and ^13^C NMR data, see Table 1.

Sclerotioloid B (**2**): yellow powder; UV (MeOH) *λ*_max_ (log *ε*) 219 (0.03), 288 (0.05) nm; HRESIMS (*m*/*z* 337.1160 [M+ Na]^+^, calcd for 337.1164); IR (KBr) *ν*_max_ 2361, 1796, 1701, 1522, 1418, 1264 cm^−1^; ^1^H and ^13^C NMR data, see Table 2.

X-ray Crystallographic Analysis of Compounds **2**. Colorless crystals of **2** suitable for X-ray diffraction were obtained from 95% MeOH (H_2_O) by slow evaporation. The crystal data were collected at 293 K using an Agilent Gemini Ultra (Agilent, PA, USA) diffractometer with Cu Kα radiation (λ = 1.54184 Å). The structure was solved using CrysAlis^Pro^ version 1.171.41.121 (Rigaku Oxford Diffraction, 2021). Empirical absorption correction using spherical harmonics implemented in SCALE3 ABSPACK scaling algorithm.

Crystal Data for **2**. C_17_H_19_N_2_O_4_, *M*r = 314.33, triclinic, space group *P* − 1 with *a* = 8.8982(11) Å, *b* = 9.6133(10) Å, *c* = 10.9564(14) Å, *α* = 67.791(11), *β* = 71.857(11), *γ* = 82.76(1), *V* = 824.51(19) Å3, *Z* = 2, *D*x = 1.266 g/cm^3^, *μ* (Cu Kα) = 0.752 mm^−1^, and *F* (000) = 332. Crystal dimensions: 0.12 × 0.12 × 0.11 mm^3^. Independent reflections: 1915 (R_int_ = 0.0576). The final R1 value was 0.0576.

Sclerotioloid C (**3**): white powder; [*α*]^20^_D_ = −11.3° (*c* 0.01, MeOH); UV (MeOH) *λ*_max_ (log *ε*) 226 (0.01), 318 (0.01) nm; HRESIMS (*m*/*z* 323.1370 [M+K]^+^, calcd for 323.1162); IR (KBr) *ν*_max_ 2923, 2824, 1735, 1512, 1455, 1253, 1179 cm^−1^; ^1^H and ^13^C NMR data, see Table 3.

### 3.6. Biological Assay

Cytotoxic activity against human cancer cell lines was evaluated following the CCK-8 assay [29]. Twenty cell lines were used, including human lung cancer cells (CCL-185), human breast cancer cells (HTB-22), human gastric cancer cells (TCP-1008), human colon cancer cells (CCL-247EMT), human hepatocellular carcinoma cells (HB-8065), human cervical cancer cells (CCL-2), human chronic myeloid leukemia cells (CRL-3344), human brain tumor cells (CRL-2020), normal human liver cells (CRL-2254), human renal clear cell adenocarcinoma cells (CRL-1932), human esophageal carcinoma cells (PDM-246), human bladder cancer cells (HTB-9), human prostate cancer cells (HTB-81), human thyroid cancer cells (CRL-3354), human pancreatic cancer cells (CRL-2151), human osteosarcoma cells (CRL-1543), human malignant melanoma cells (CRL-1619), human rhabdomyosarcoma cells (CRL-1598), human embryonic kidney cells (CRL-3216) and human gallbladder carcinoma cells (PDM-273). Adriamycin hydrochloride was used as a positive control.

Antibacterial activity was evaluated following the standards recommended by Pierce [30], with ampicillin sodium as a positive control. The antifungal bioassay was conducted following the standards recommended by the Clinical and Laboratory Standards Institute [30], with amphotericin B used as a positive control.

Compounds **1**–**9** were tested against 20 pathogenic bacteria, including *Enterobacter cloacae*, *E. hormaechei*, *Aeromonas salmonicida*, *Escherichia coli*, *Pseudomonas fulva*, *Photobacterium halotolerans*, *P*. *aeruginosa*, *Staphylococcus aureus*, *Bacillus subtilis*, *Vibrio Parahemolyticus* HUB183, *V. alginolyticus* HUB184, *V. anguillarum* HUB185, *S*. *aureus* ATCC 43300, *Enterococcus faecalis* ATCC 51299, *E*. *faecium* ATCC 35667, *S*. *aureus* ATCC 33591, *S*. *aureus* ATCC 29213, *S*. *aureus* ATCC 25923, *V*. *vulnificus* MCCC E1758, *V*. *campbellii* MCCC E333, and two pathogenic fungi *Candida albicans* ATCC 24,433 and *C. parapsilosis* ATCC 22019.

The DPPH scavenging assay was performed using the method described by Aquino et al. [31]. The reaction mixture consisted of freshly prepared DPPH dissolved in ethanol (100 µmol/L) mixed with different concentrations of the tested compound. The reaction mixture was incubated for 20 min at room temperature in the dark, and the optical density was recorded at 517 nm.

The bioassay for NO production inhibitory activity was conducted as described by Xia et al. [32]. The mouse macrophages were seeded in 96-well plates. In each well, LPS (1 μg/mL) was added after treating with or without the tested compound for 24 h. The NO production in the supernatant was detected by the Griess reaction. The absorbance at 540 nm was measured with a microplate reader. The NO concentration and the inhibitory rate were calculated as a calibration curve. Dexamethasone was used as the positive control. Experiments were performed in triplicate, and the data are described as mean ± SD of three independent experiments.

## 4. Conclusions

In conclusion, nine alkaloids, including three new ones, were obtained by using MS/MS-based molecular networking for continuous investigation of the marine-derived fungus, *A*. *sclerotiorum* ST0501. Sclerotioloid A (**1**) represents the first example of 2,5-diketopiperazine alkaloid with a rare terminal alkyne. Additionally, the absolute configuration of compound **2** was unambiguously determined by single crystal X-ray analysis. Compound **2** showed the inhibition of NO production induced by lipopolysaccharide (LPS). Compound **3** displayed weak proliferation inhibitory activity against human chronic myeloid leukemia cells K-562 and human renal clear cell adenocarcinoma cell 786-O. Therefore, the potential of the marine-derived fungus, *A*. *sclerotiorum* ST0501, to produce novel bioactive secondary metabolites is worthy of further exploration.

## Figures and Tables

**Table 1:** NMR data for compound **1** (DMSO*-d*_6_, 400 MHz/100 MHz).

Position	*δ*_C_, mult.	Δ_H_, mult. (*J* in Hz)	COSY	HMBC
1	NH	8.42 (s)	H-6	C-3
2	163.4, C			C-6, C-10
3	129.9, C			1-NH, C-7
4	N			
5	165.7, C			C-6, C-7
6	44.5, CH_2_	4.01, d, (2.4)	1-NH	C-2, C-5
7	32.9, CH_2_	4.13, d, (2.4)		C-3, C-5, C-9
8	78.2, C			
9	74.5, CH	3.10, t, (2.4)		C-7
10	120.8, CH	7.10 (s)		C-2, C-16
11	133.4, C			C-16
12	128.6, CH	7.35–7.40 (m)	H-13	
13	129.4, CH	7.35–7.40 (m)	H-12, H-14	
14	128.8, CH	7.35–7.40 (m)	H-13, H-15	
15	129.4, CH	7.35–7.40 (m)	H-14, H-16	
16	128.6, CH	7.35–7.40 (m)	H-15	C-10, C-11

**Table 2:** NMR data for compound **2** (DMSO-*d*_6_, 600 MHz/150 MHz).

Position	*δ*_C_, mult.	Δ_H_, mult. (*J* in Hz)	COSY	HMBC
1	21.9, CH_3_	1.77 (s)		C-2
2	169.0, C			1-NH
3	NH	8.06 (s)	H-4	C-2
4	40.4, CH_2_	3.56, dd, (17.0, 5.7) 3.66, dd, (17.1, 5.7)	1-NH	C-5
5	168.4, C			C-4
6	N			
7	35.5, CH_2_	4.24, dd, (17.6, 2.5) 4.38, dd, (17.5, 2.5)		C-8, C-9
8	77.5, C			C-7
9	75.8, CH	3.15 (s)		C-7
10	126.6, C			
11	164.5, C			C-12, C-13
12	52.5, OCH_3_	3.81 (s)		C-11
13	139.5 CH	7.84 (s)		C-11, C-14 C-15
14	131.5, C			C-13
15	130.2, CH	7.75 (s)	H-16	C-13
16	128.8, CH	7.46, d, (7.6)	H-15, H-17	
17	131.0, CH	7.49, d, (6.9)	H-16, H-18	
18	128.8, CH	7.46, d, (7.6)	H-17, H-19	
19	130.2, CH	7.73 (s)	H-18	

**Table 3:** NMR data for compound **3** (DMSO-*d*_6_, 500 MHz/125 MHz).

Position	*δ*_C_, mult.	Δ_H_, mult. (*J* in Hz)	COSY	HMBC	NOESY
1	NH	8.35–8.37, m	H-6	C-3, C-5	H-6, H-7
2	160.7, C			4-NH, C-6, C-8	
3	125.3, C			1-NH, C-10, C-14	
4	NH	9.79, s		C-2, C-5, C-6	H-12, H-13
5	167.6, C			1-NH, 4-NH, C-6, C-7	
6	50.3, CH	4.11, d, (6.8)	1-NH, H-7	C-2, C-5, 4-NH, C-7	1-NH, H-7
7	19.2, CH_3_	1.33, d, (7.0)	H-6	C-5, C-6	1-NH, H-6
8	114.3, CH	6.64 (s)		C-2, C-10	
9	125.7, C				
10	130.8, CH	7.44, d, (8.8)	H-14	C-8, C-12, C-14	
11	114.8, CH	6.95, d, (8.8)	H-13	C-3, C-12, C-13	H-17
12	158.2, C			C-10, C-11, C-13, C-14, C-17	4-NH
13	114.8, CH	6.95, d, (8.8)	H-11	C-3, C-11, C-12	H-17
14	130.8, CH	7.44, d, (8.8)	H-10	C-10, C-12	
15	137.3, C			C-17, C-18, C-19	
16	119.8, CH	5.43, tt, (6.7, 1.4)	H-17	C-17, C-18, C-19	H-17, H-18
17	64.3, CH_2_	4.55, d, (6.7)	H-16, H-18	C-12, C-15, C-16	H-11, H-13, H-16
18	18.0, CH_3_	1.71, d, (1.3)	H-17	C-15, C-16	H-16
19	25.4, CH_3_	1.74 (s)		C-15, C-16	

## Supplementary Materials

The following supporting information can be downloaded at: https://www.mdpi.com/article/10.3390/md21040219/s1, Figures S1–S19: ^1^H NMR, ^13^C NMR, ^1^H-^1^H COSY, HSQC, HMBC, HRESIMS, UV and IR spectra of compounds **1** and **2**. Figure S20–S28: ^1^H NMR, ^13^C NMR, ^1^H-^1^H COSY, HSQC, HMBC, NOESY, HRESIMS, UV and IR spectra of compound **3**. Table S1: The activity results about anti-tumor of compound **3**. Table S2: The activity results about anti-inflammatory of compounds **1**–**3**.
