# Supplementary material for: Sclerotioloids A–C: Three New Alkaloids from the Marine-Derived Fungus Aspergillus sclerotiorum ST0501
Source: Mar Drugs. 2023 Mar 29;21(4):219. doi: 10.3390/md21040219 (PMC10142895; doi:10.3390/md21040219)
Supplement: Supplementary file 1 [file marinedrugs-21-00219-s001.zip › marinedrugs-2238365-supplementary.pdf]

Sclerotioloids A−C: Three New Alkaloids from the Marine-derived Fungus *Aspergillus sclerotiorum* ST0501

List of Supporting Information

Figure S1. ^{1}H NMR spectrum of compound **1** in DMSO-d_6 (400 MHz).

Figure S2. ^{13}C NMR spectrum of compound **1** in DMSO-d_6 (100 MHz).

Figure S3. Partial ^{13}C NMR spectrum of compound **1** in DMSO-d_6 (100 MHz).

Figure S4. ^{1}H-^{1}H COSY spectrum of compound **1** in DMSO-d_6.

Figure S5. HSQC spectrum of compound **1** in DMSO-d_6.

Figure S6. HMBC spectrum of compound **1** in DMSO-d_6.

Figure S7. HRESIMS spectrum (positive ion mode) of compound **1**.

Figure S8. ^{1}H NMR spectrum of compound **2** in DMSO-d_6 (600 MHz).

Figure S9. Partial ^{1}H NMR spectrum of compound **2** in DMSO-d_6 (600 MHz).

Figure S10. ^{13}C NMR spectrum of compound **2** in DMSO-d_6 (150 MHz).

Figure S11. Partial ^{13}C NMR spectrum of compound **2** in DMSO-d_6 (150 MHz).

Figure S12. ^{1}H-^{1}H COSY spectrum of compound **2** in DMSO-d_6.

Figure S13. HSQC spectrum of compound **2** in DMSO-d_6.

Figure S14. HMBC spectrum of compound **2** in DMSO-d_6.

Figure S15. HRESIMS spectrum (positive ion mode) of compound **2**.

Figure S16. ^{1}H NMR spectrum of compound **3** in DMSO-d_6 (500 MHz).

Figure S17. ^{13}C NMR spectrum of compound **3** in DMSO-d_6 (125 MHz).

Figure S18. ^{1}H-^{1}H COSY spectrum of compound **3** in DMSO-d_6.

Figure S19. HSQC spectrum of compound **3** in DMSO-d_6.

Figure S20. HMBC spectrum of compound **3** in DMSO-d_6.

Figure S21. NOESY spectrum of compound **3** in DMSO-d_6.

Figure S22. HRESIMS spectrum (positive ion mode) of compound **3**.

Table S1. The activity results about antitumor of compound **3**.

Table S2. The activity results about anti-inflammatory of compounds **1**−**3**.

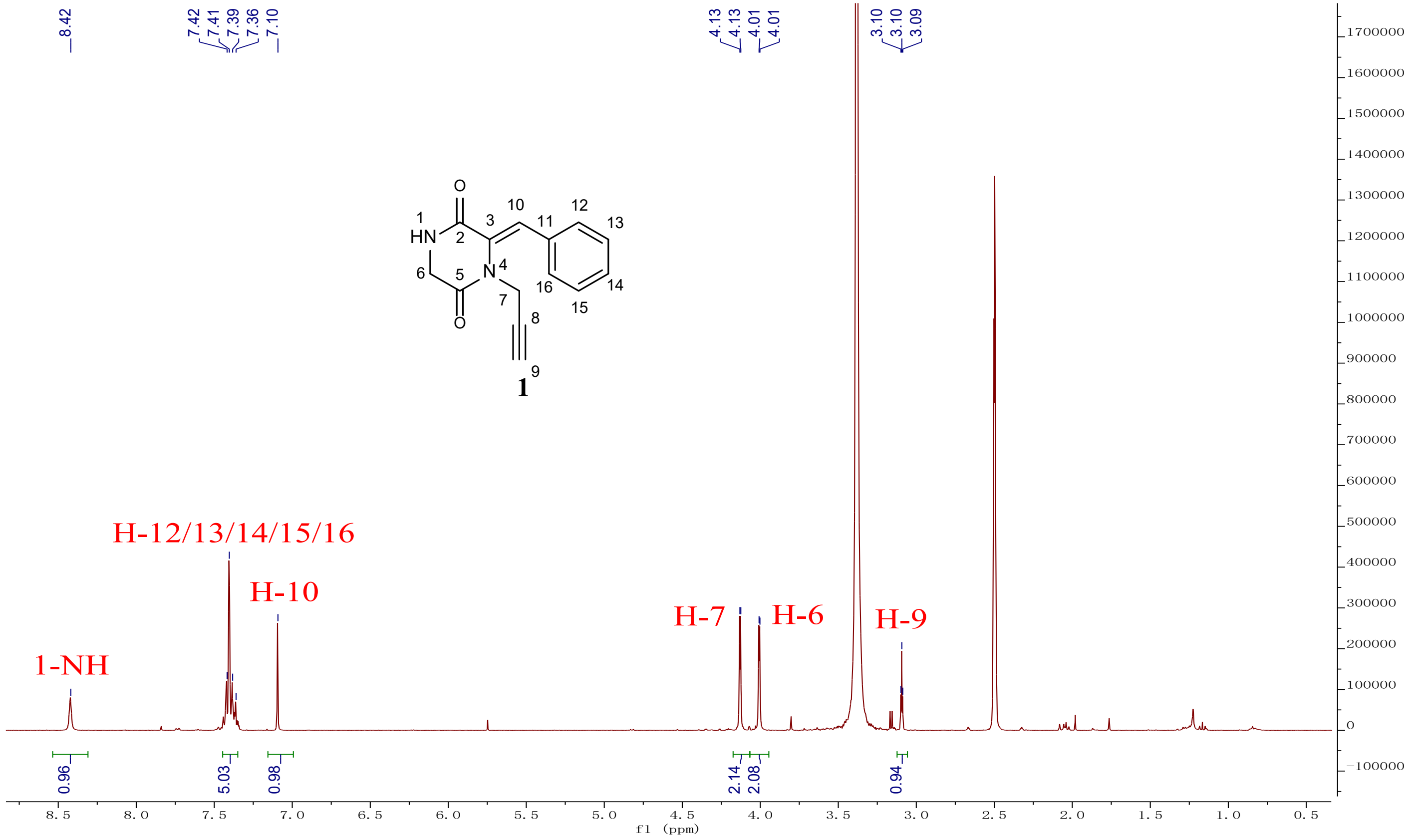

Figure S1. ^{1}H NMR spectrum of compound **1** in DMSO-d_6 (400 MHz).

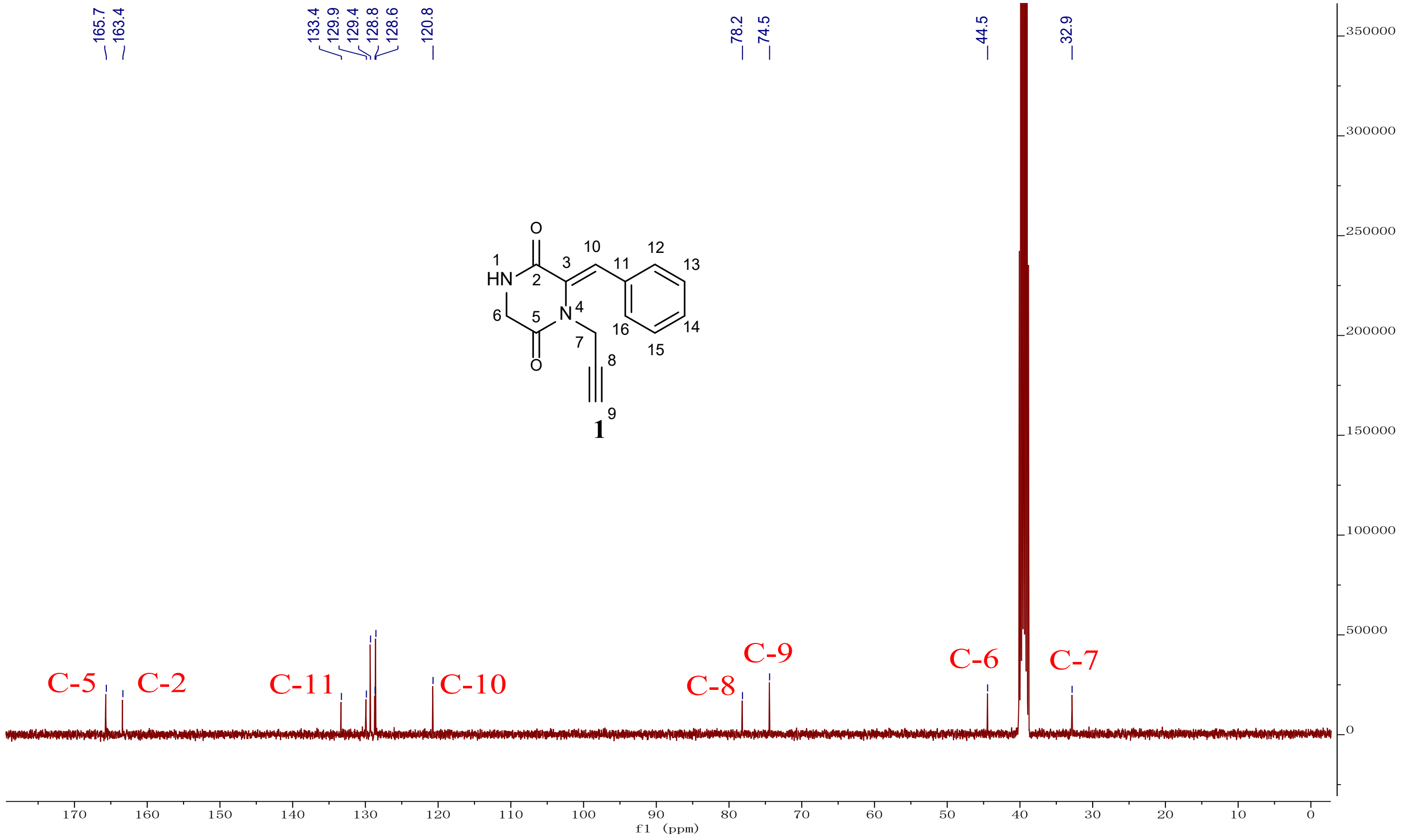

Figure S2. ^{13}C NMR spectrum of compound **1** in DMSO-d_6 (100 MHz).

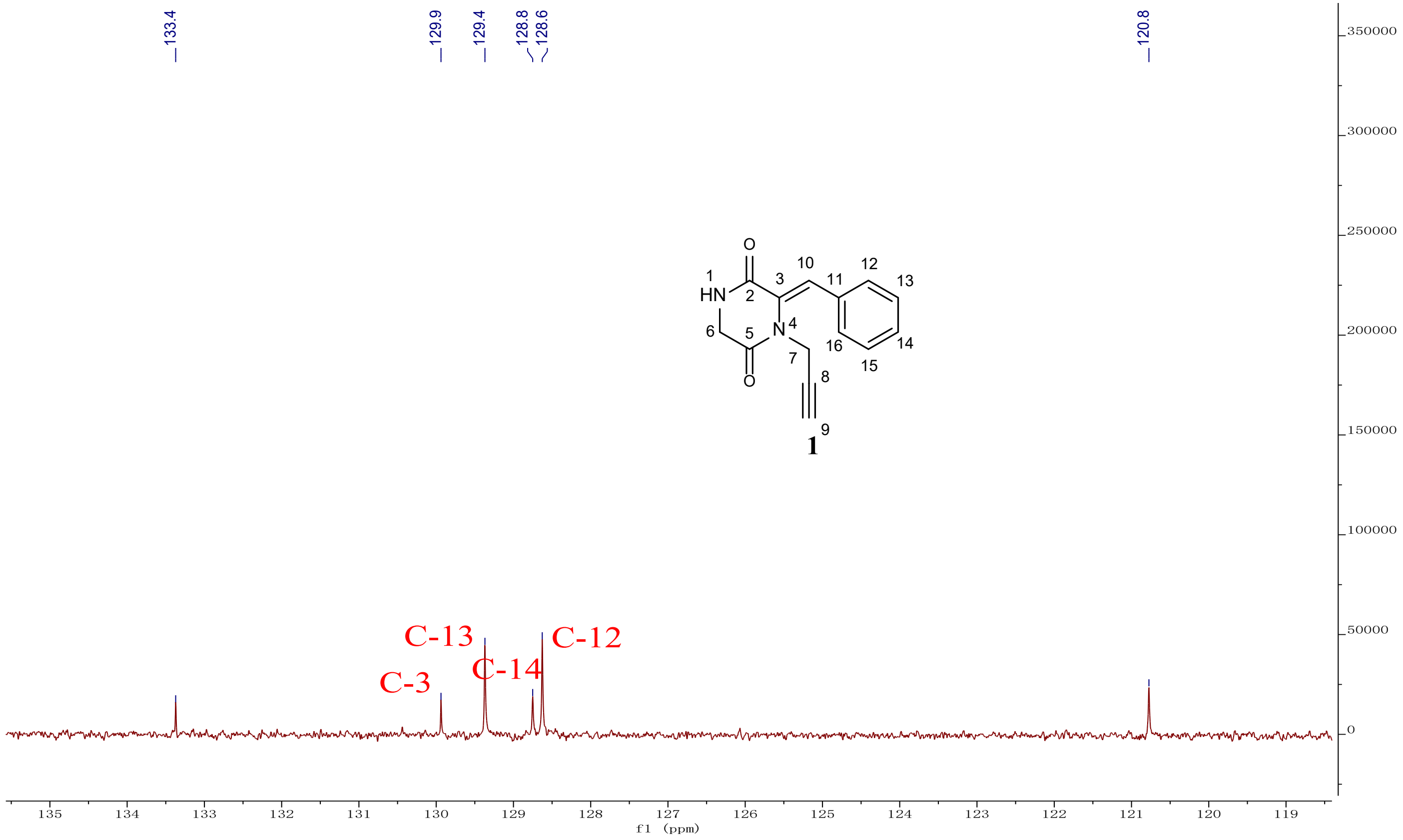

Figure S3. Partial ^{13}C NMR spectrum of compound **1** in DMSO-d_6 (100 MHz).

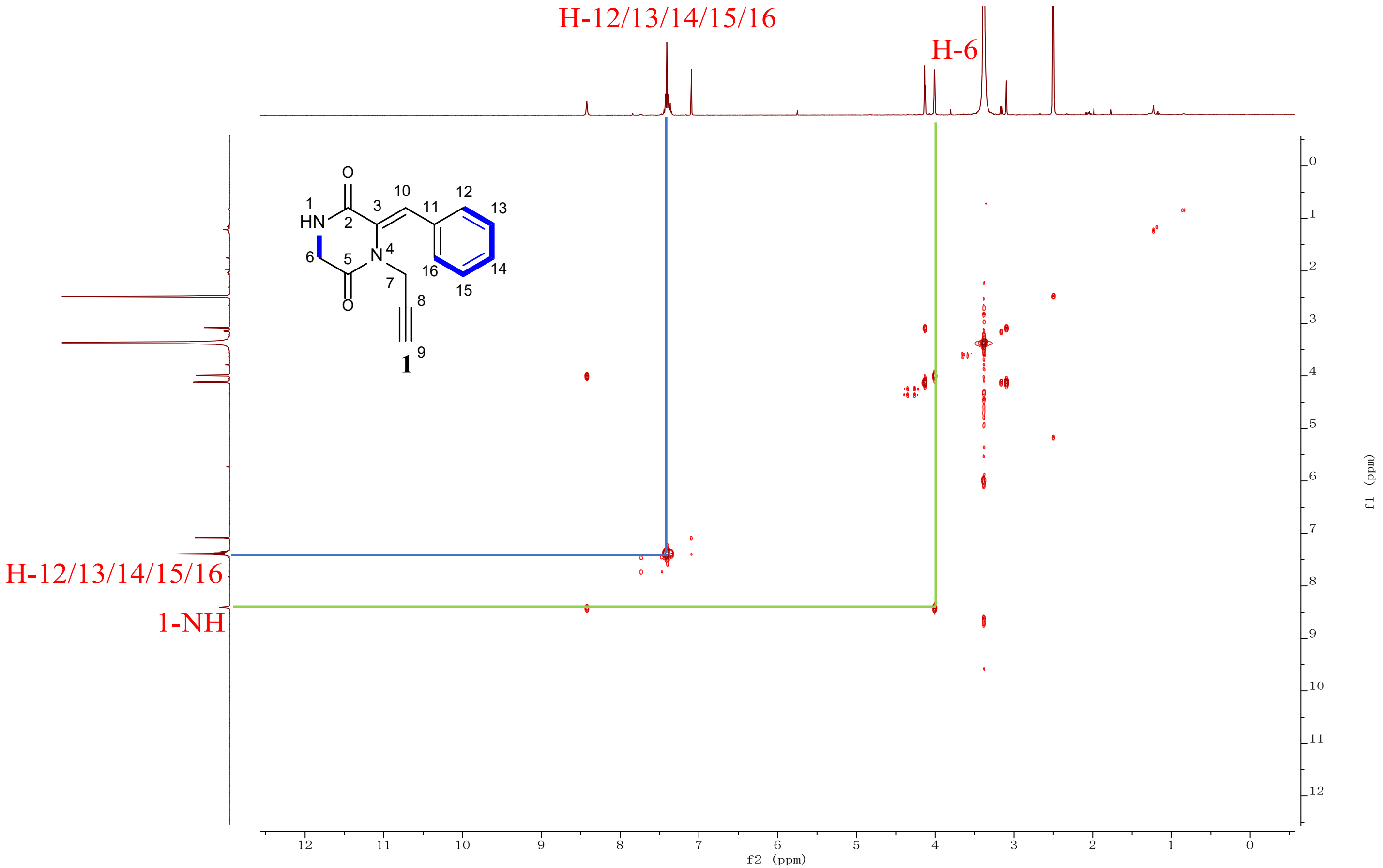

Figure S4. ^{1}H-^{1}H COSY spectrum of compound **1** in DMSO-d_6.

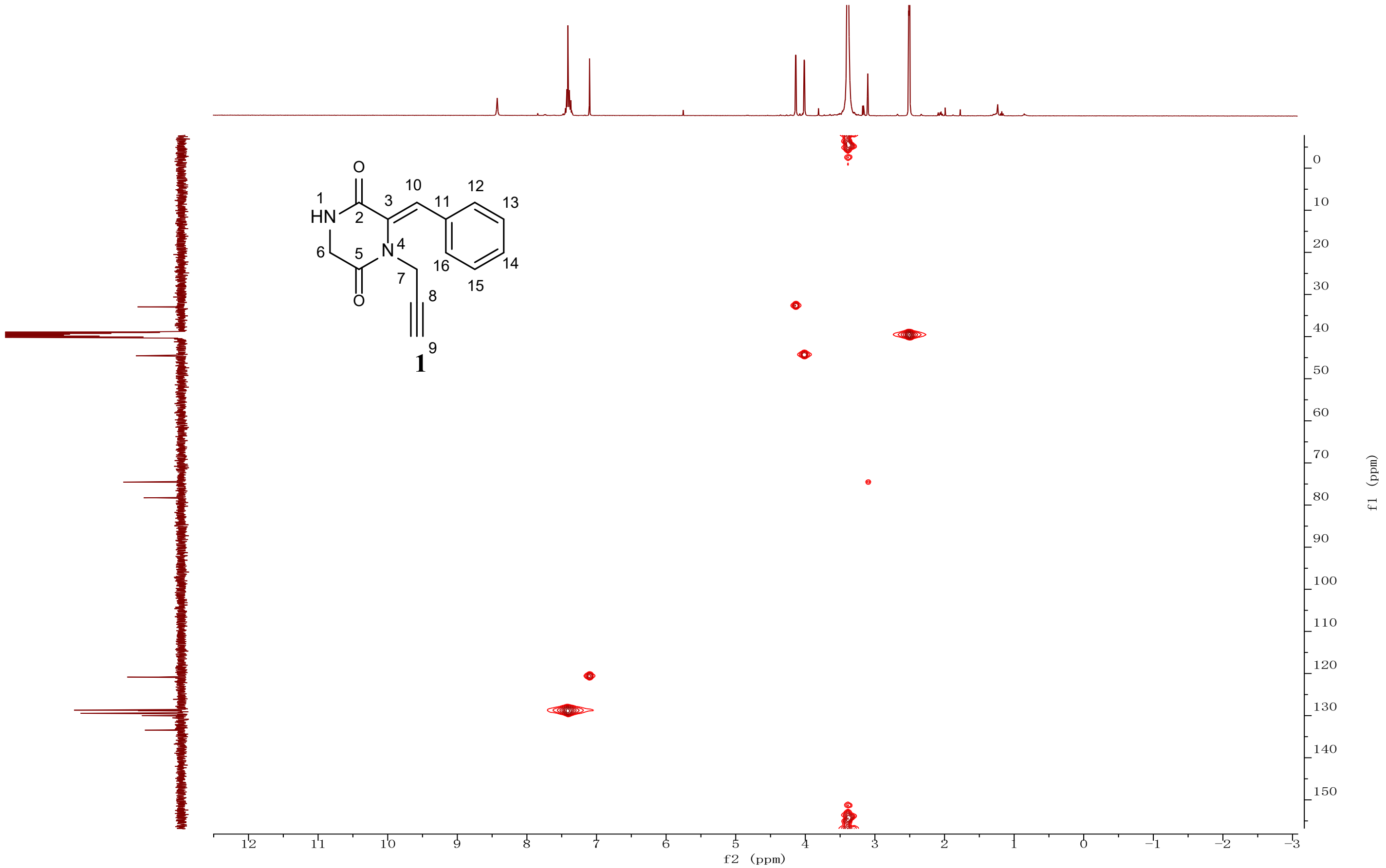

Figure S5. HSQC spectrum of compound **1** in DMSO-d_6.

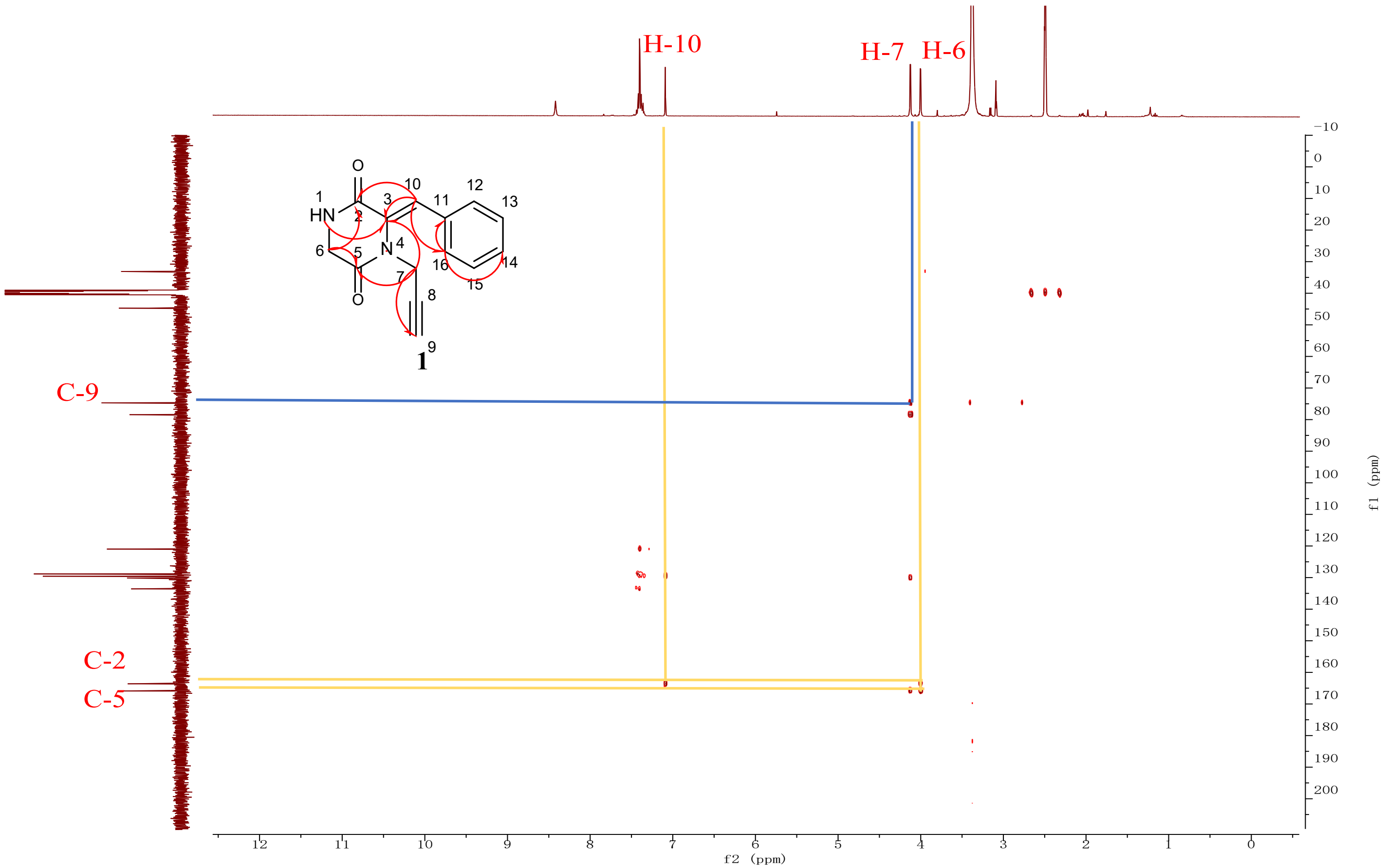

Figure S6. HMBC spectrum of compound **1** in DMSO-d_6.

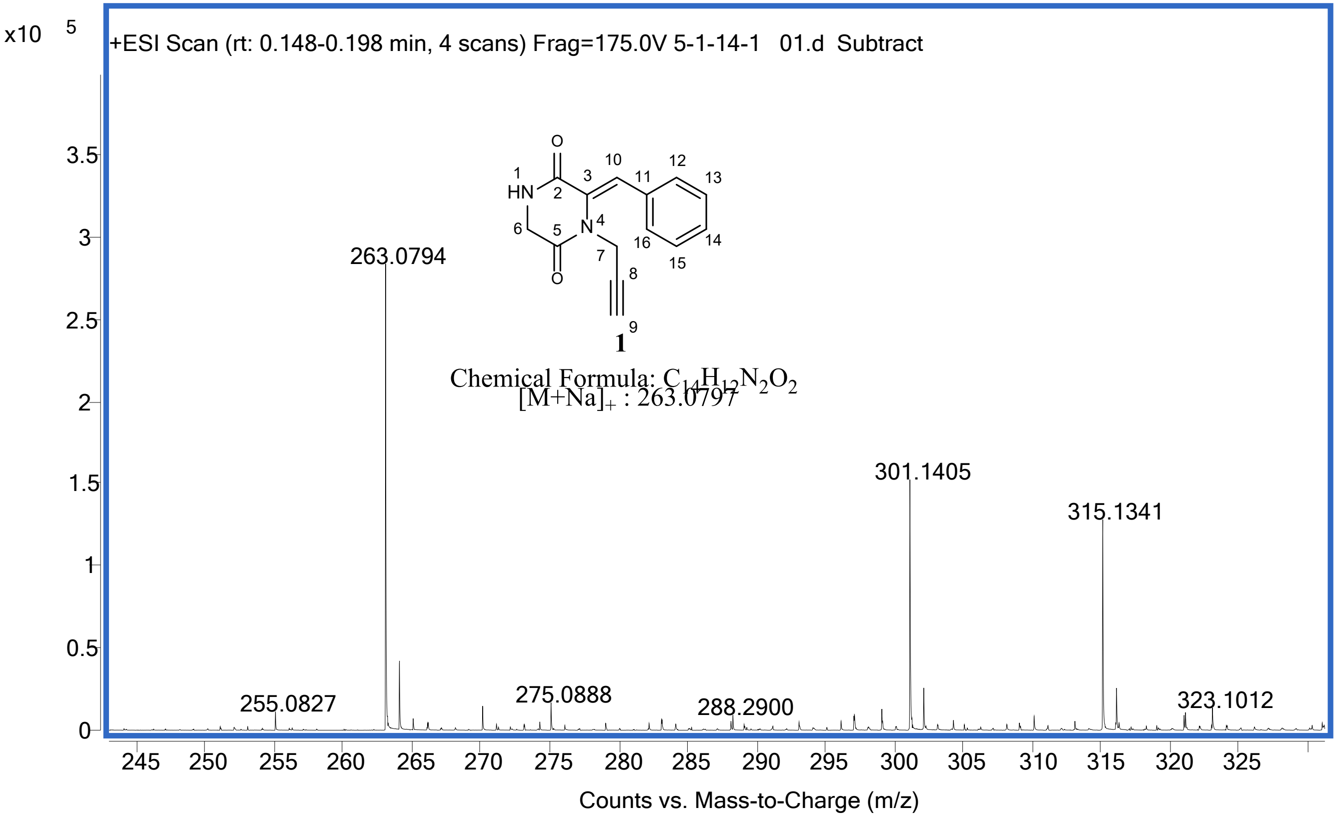

Figure S7. HRESIMS spectrum (positive ion mode) of compound **1**.

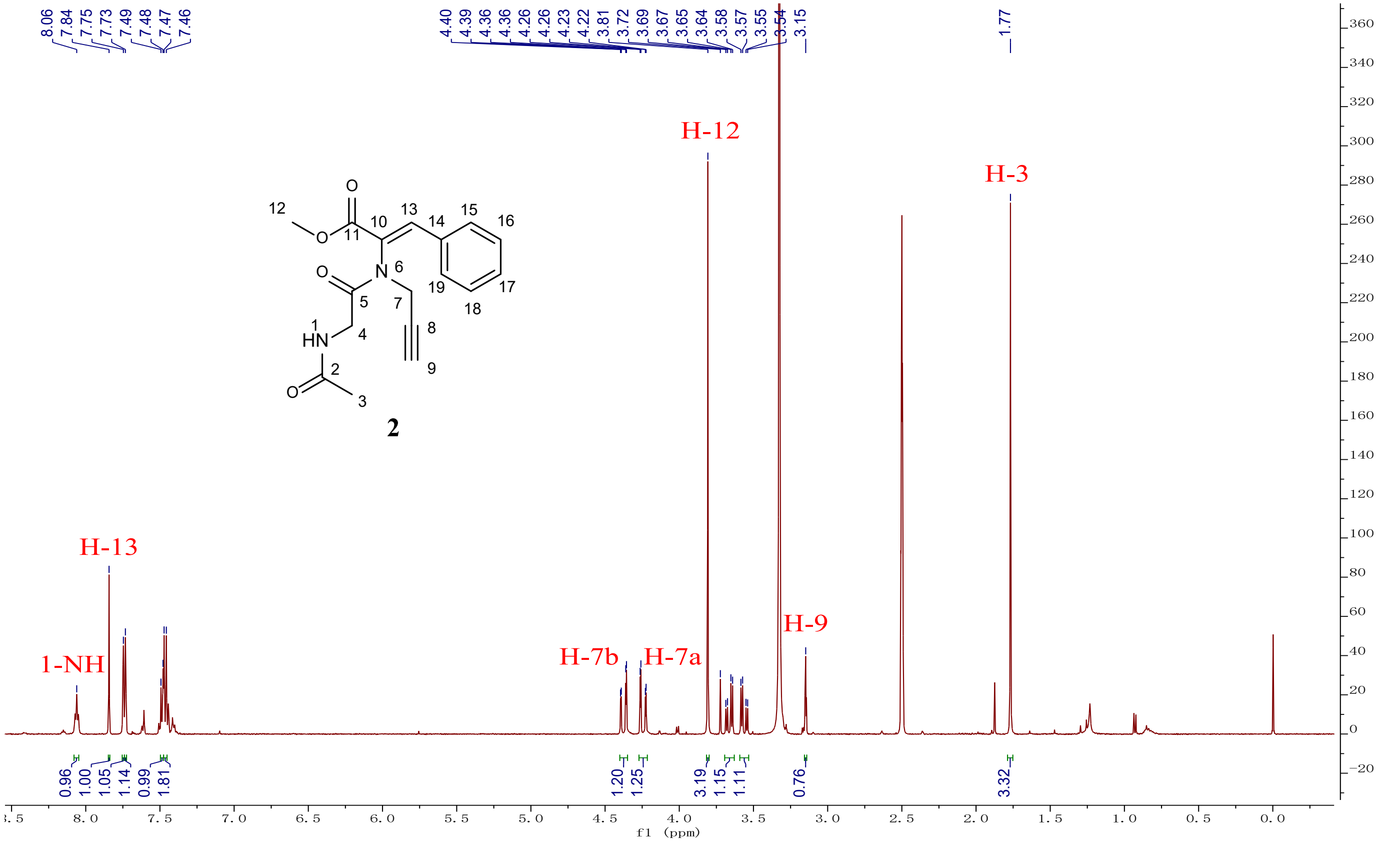

Figure S8. ^{1}H NMR spectrum of compound **2** in DMSO-d_6 (600 MHz).

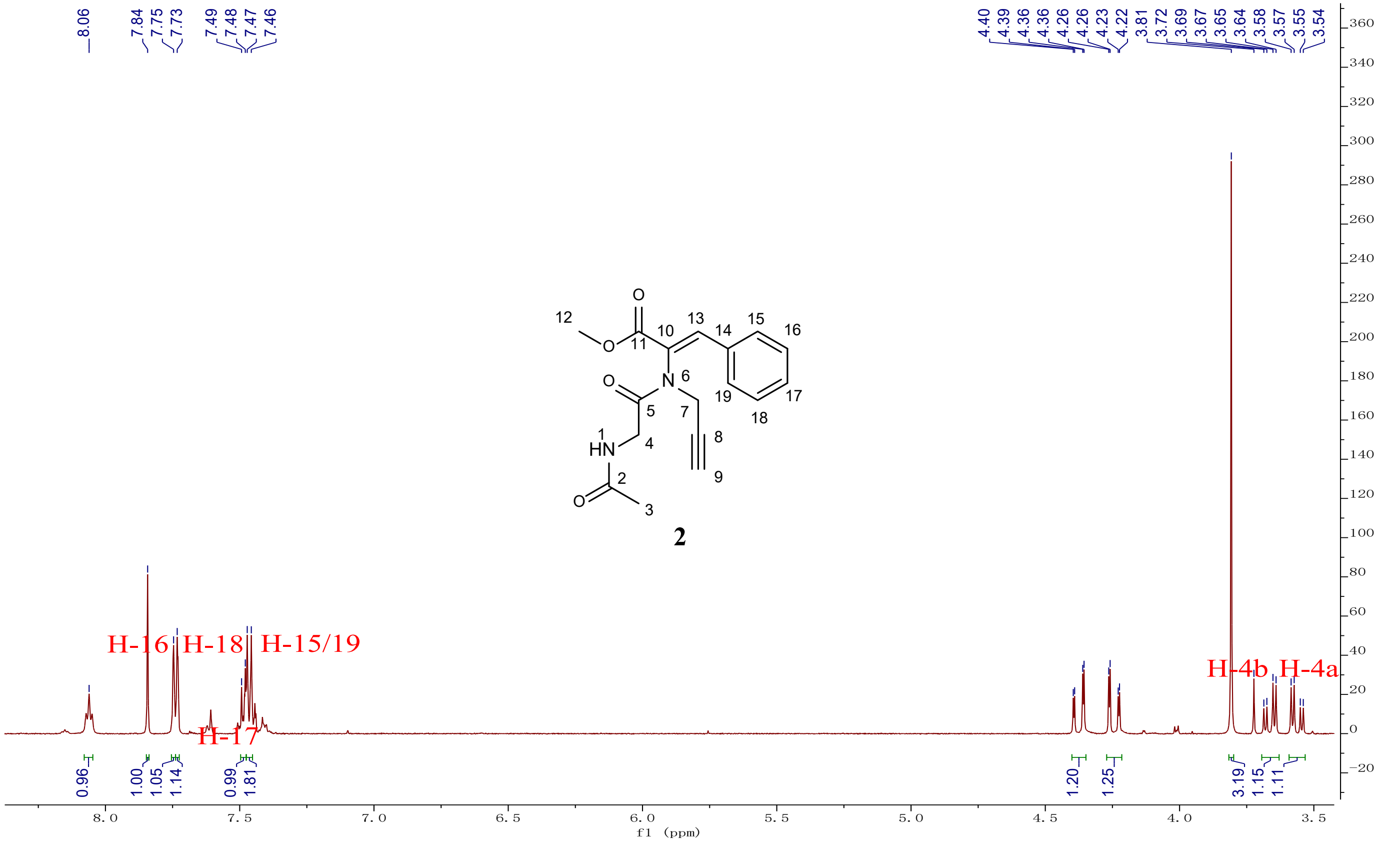

Figure S9. Partial ^{1}H NMR spectrum of compound **2** in DMSO-d_6 (600 MHz).

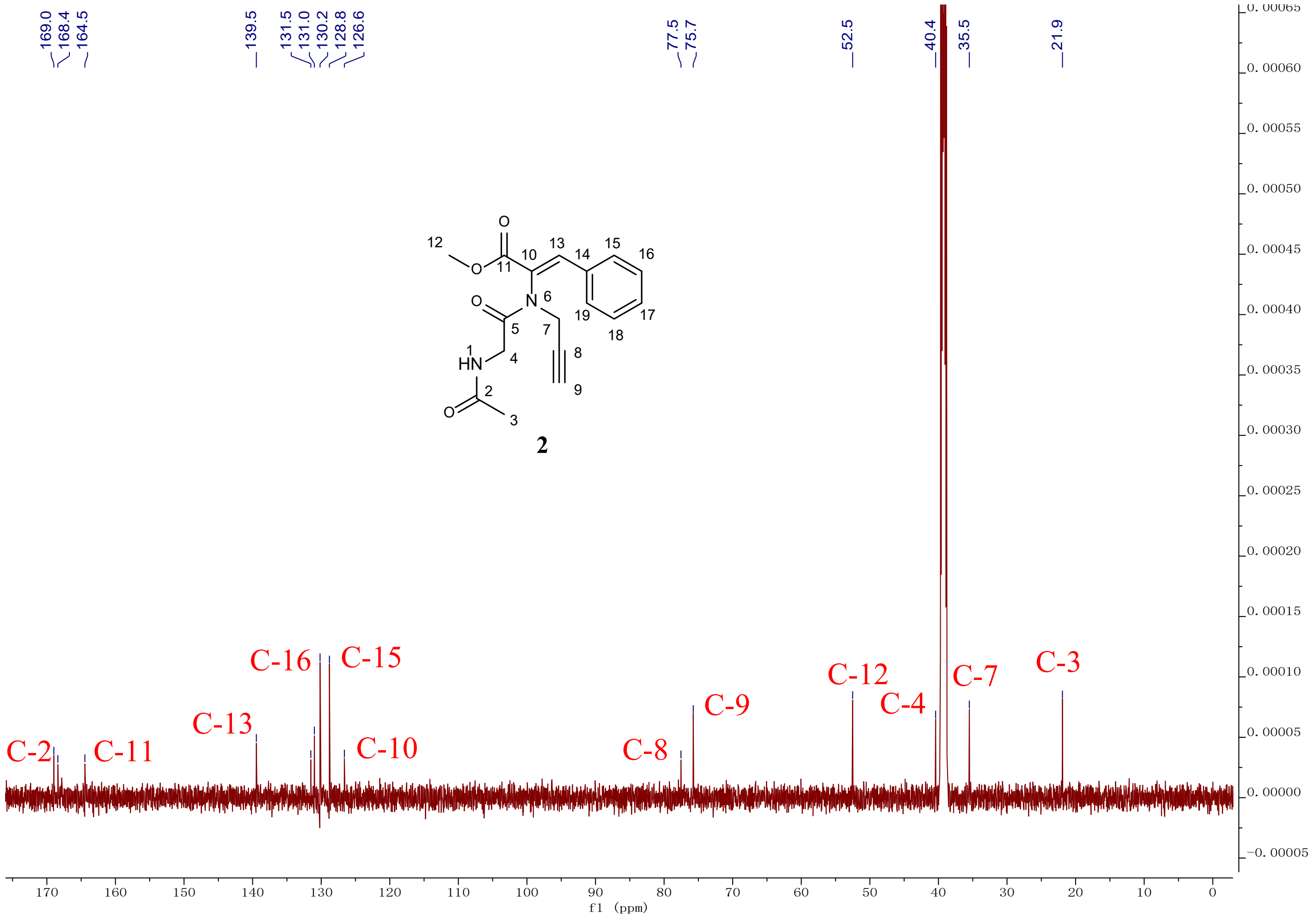

Figure S10. ^{13}C NMR spectrum of compound **2** in DMSO-d_6 (150 MHz).

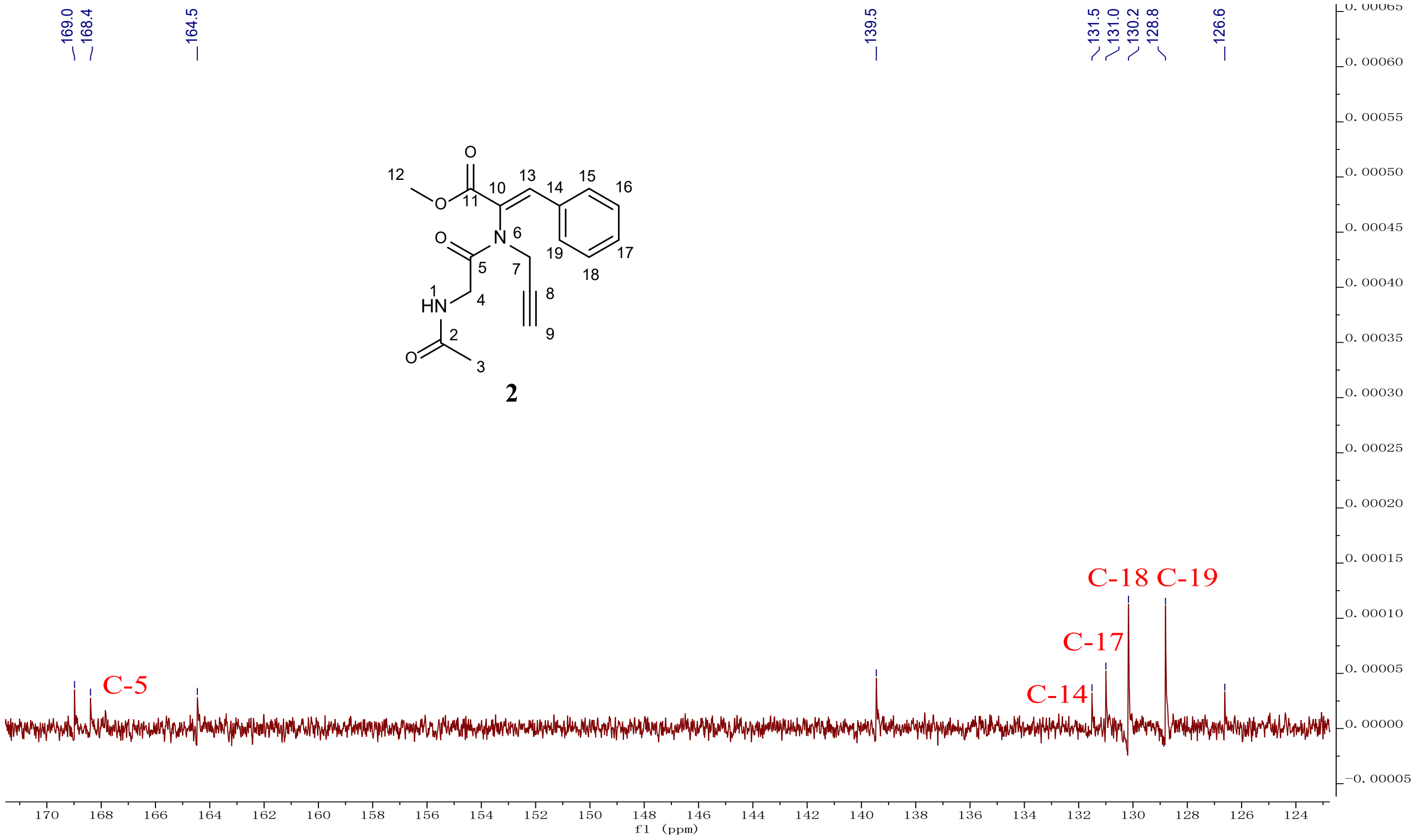

Figure S11. Partial ^{13}C NMR spectrum of compound **2** in DMSO-d_6 (150 MHz).

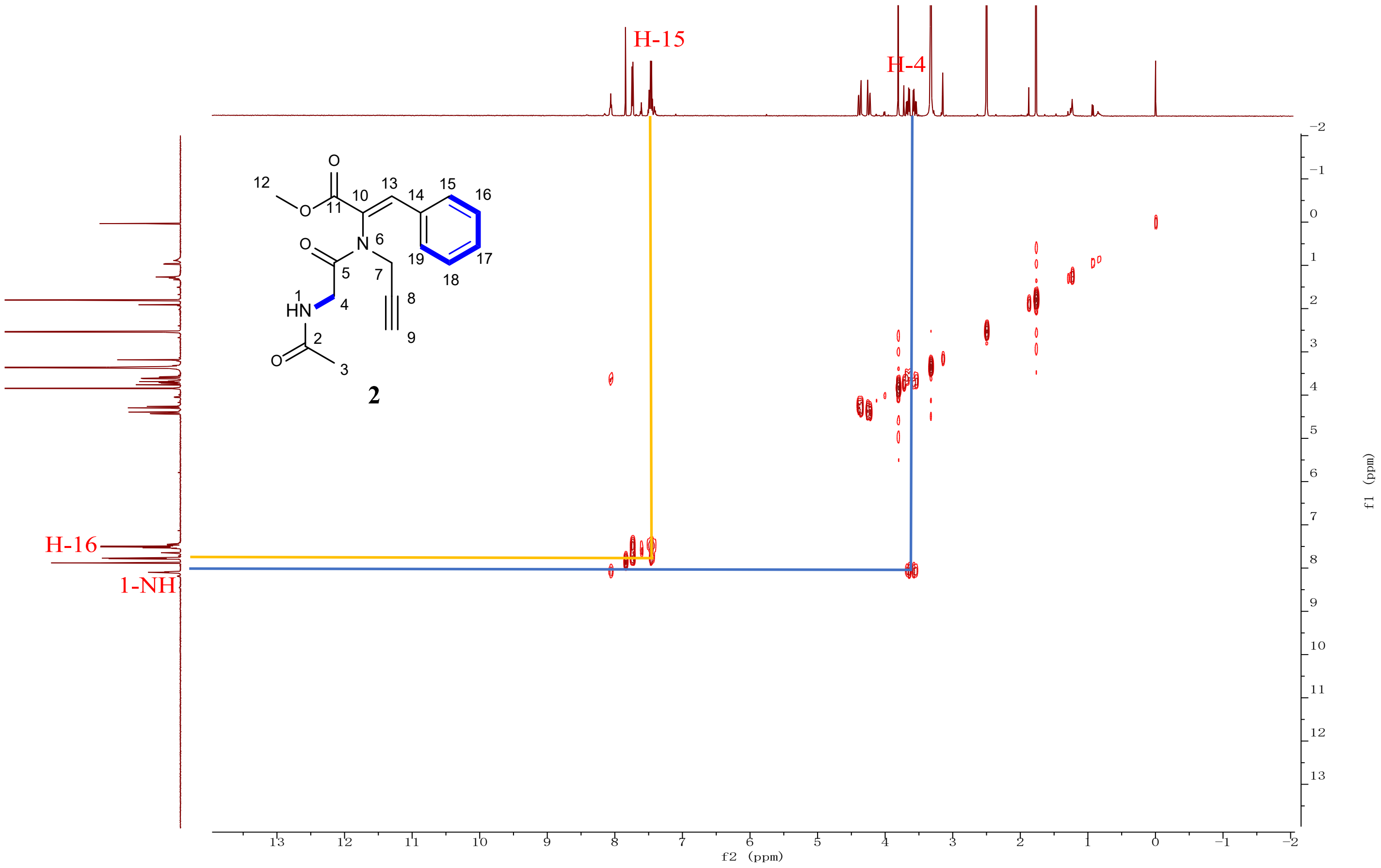

Figure S12. ^{1}H-^{1}H COSY spectrum of compound **2** in DMSO-d_6.

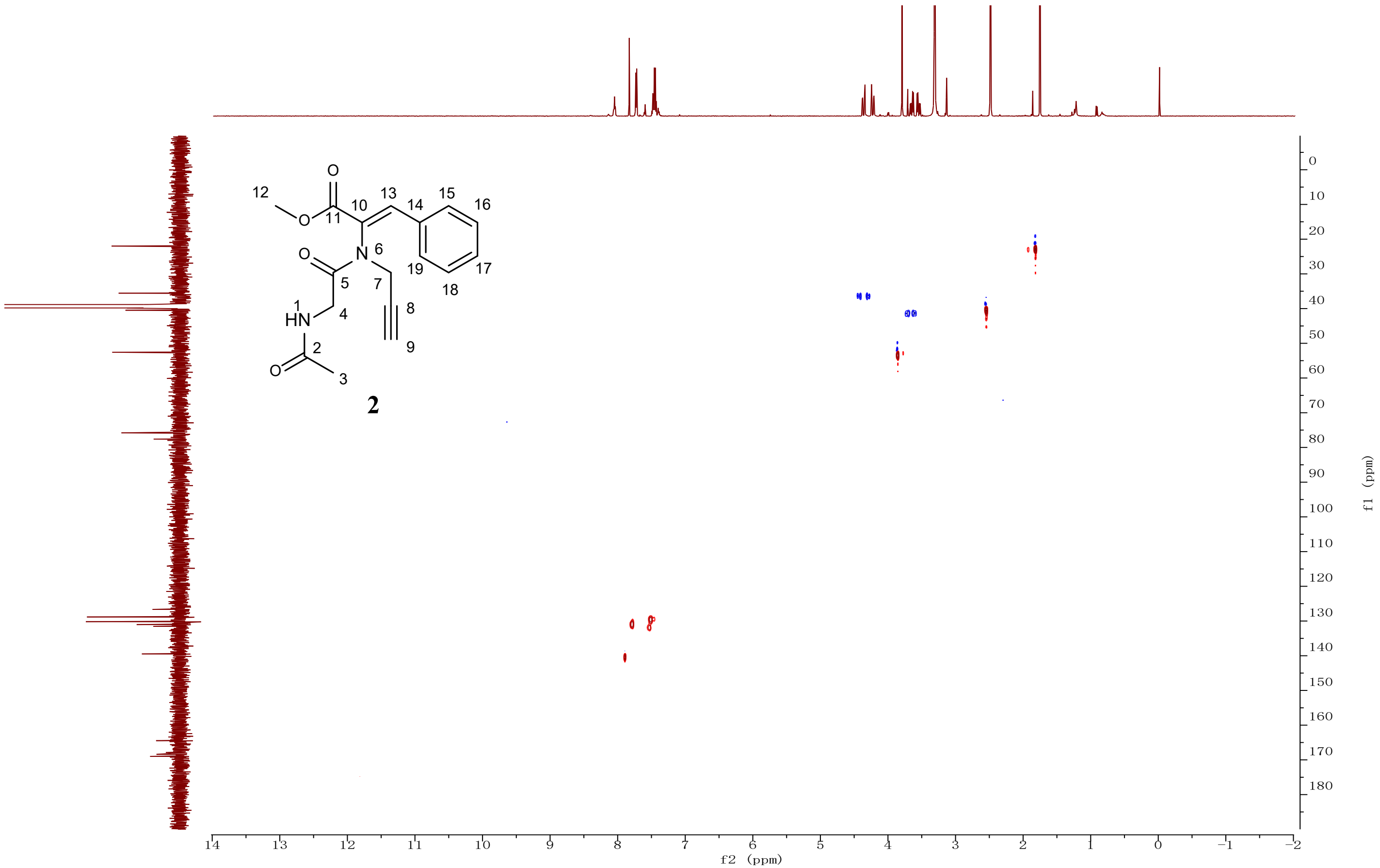

Figure S13. HSQC spectrum of compound **2** in DMSO-d_6.

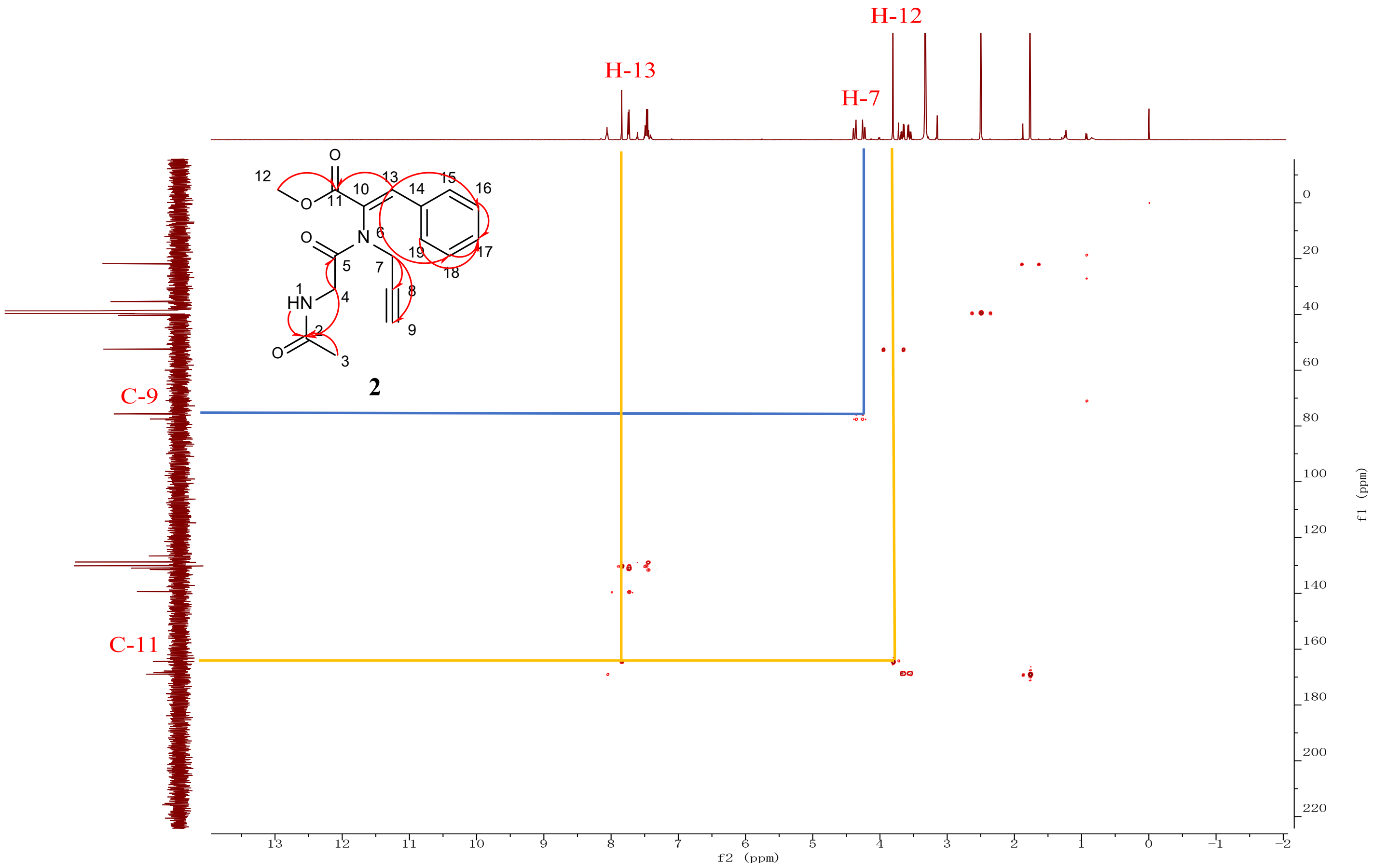

Figure S14. HMBC spectrum of compound **2** in DMSO-d_6.

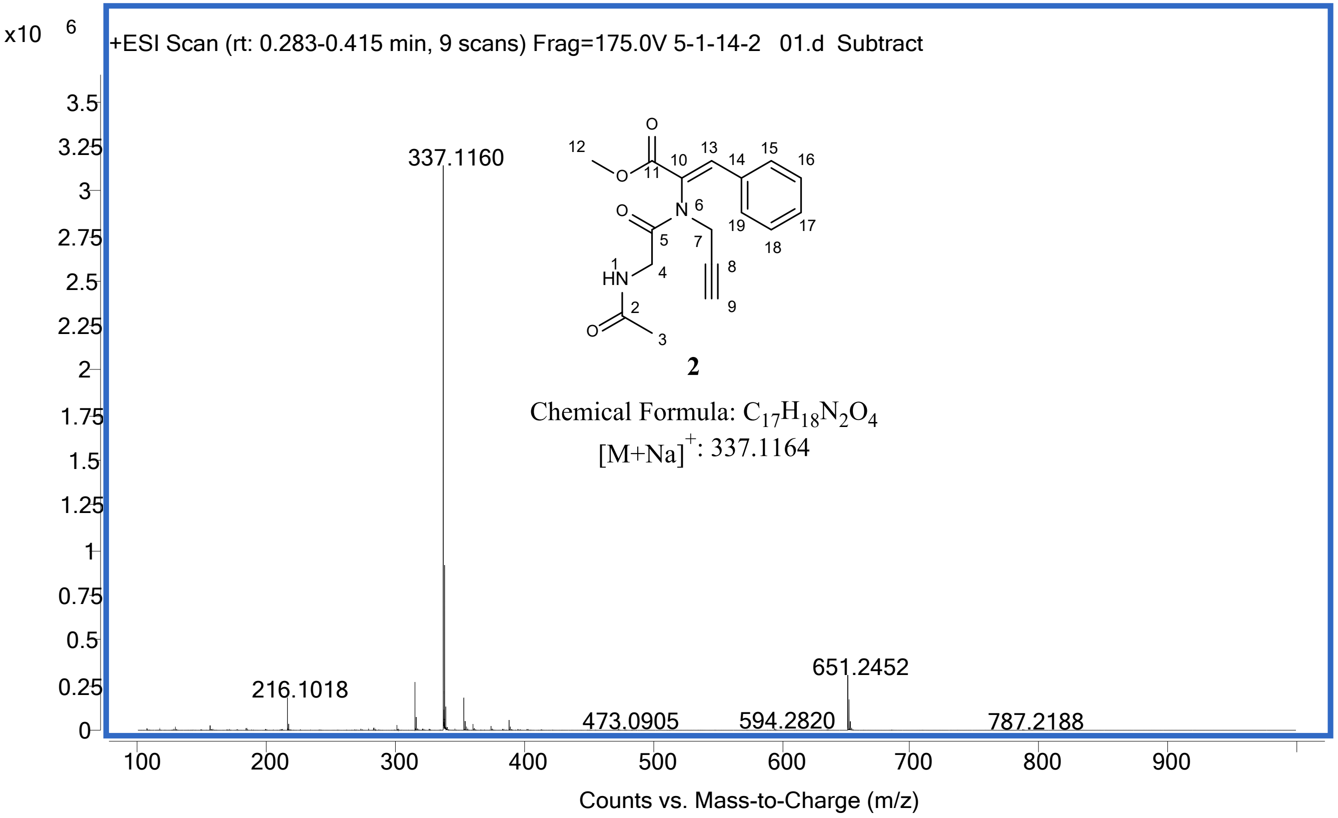

Figure S15. HRESIMS spectrum (positive ion mode) of compound **2**.

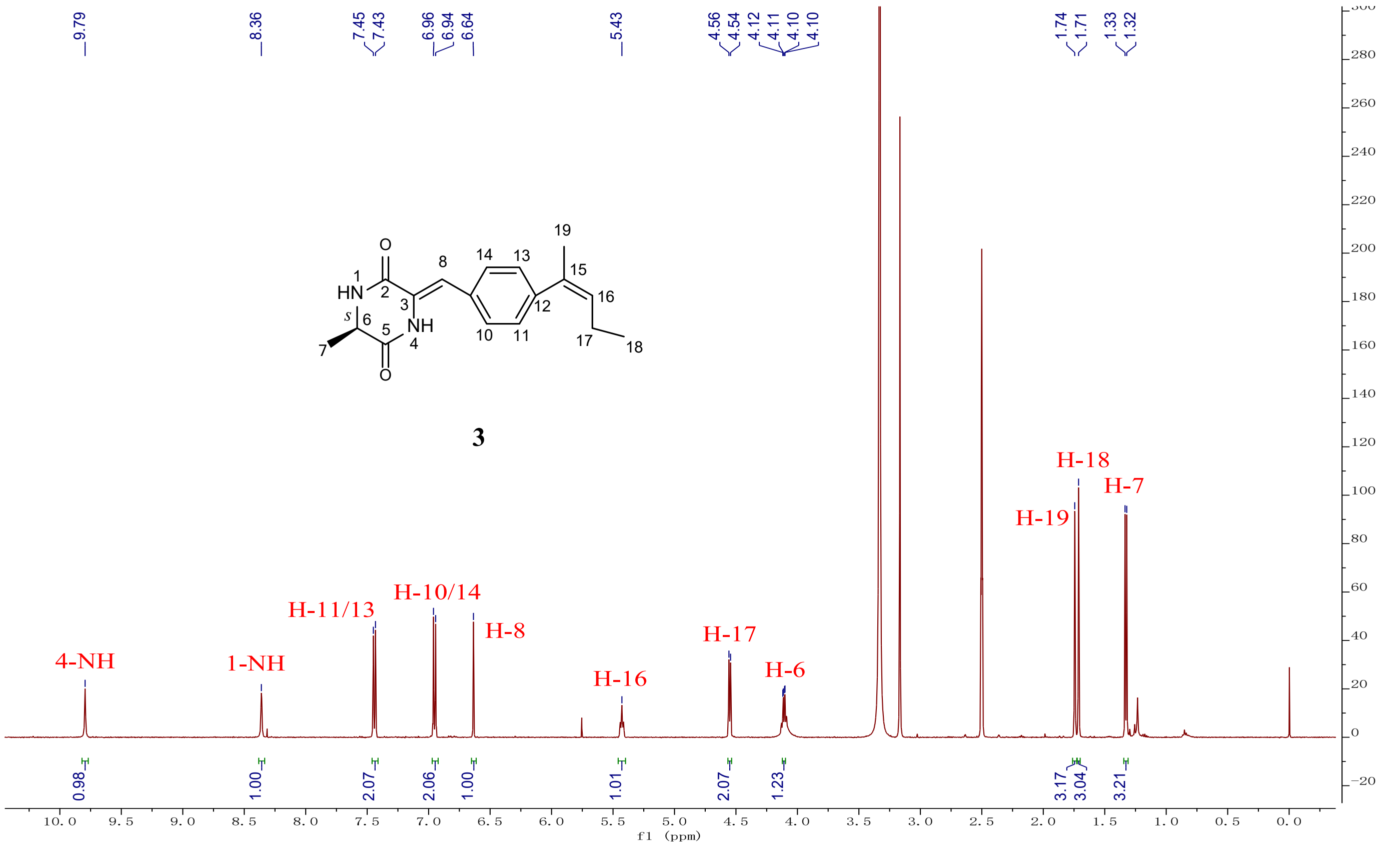

Figure S16. ^{1}H NMR spectrum of compound **3** in DMSO-d_6 (500 MHz).

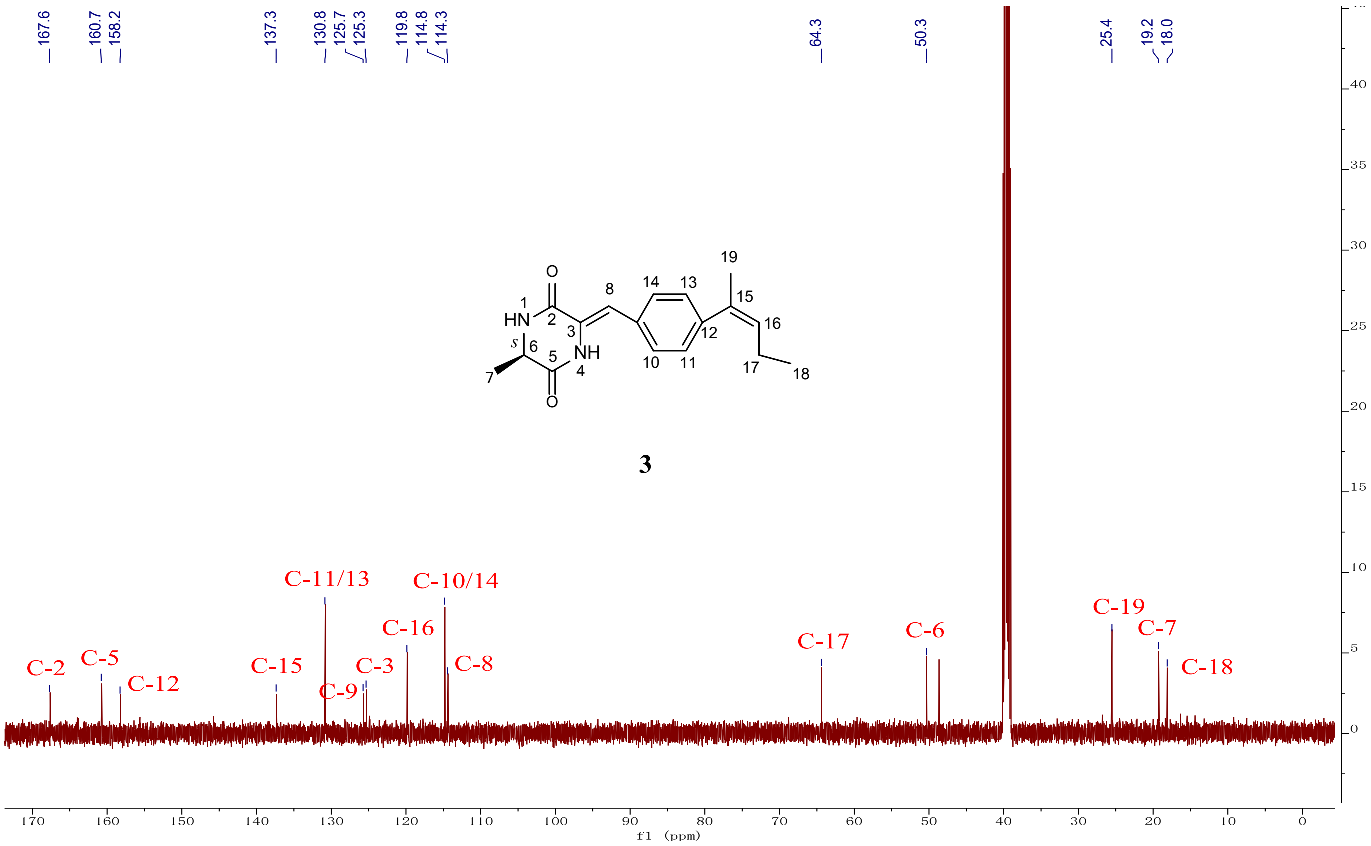

Figure S17. ^{13}C NMR spectrum of compound **3** in DMSO-d_6 (125 MHz).

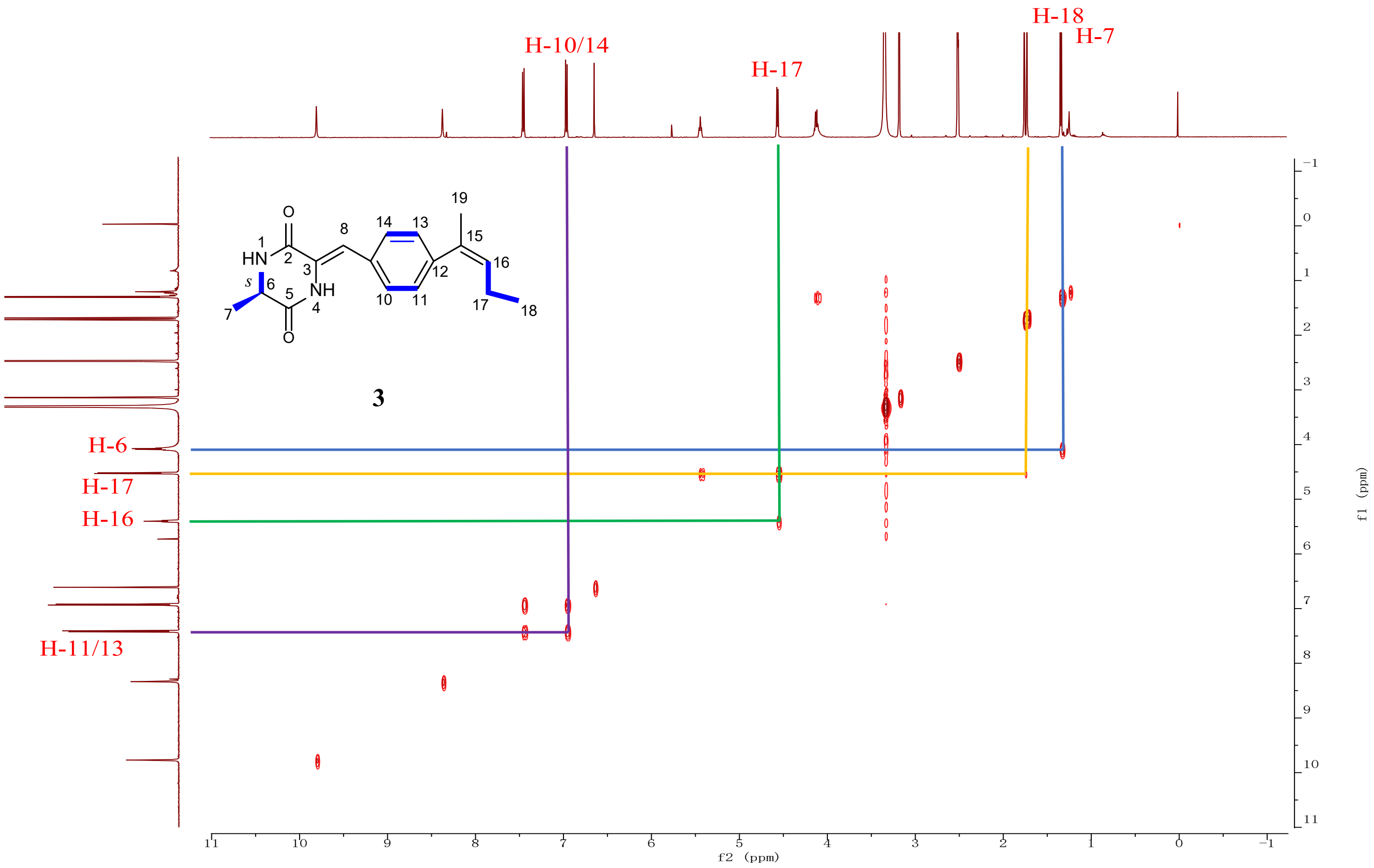

Figure S18. ^{1}H-^{1}H COSY spectrum of compound **3** in DMSO-d_6.

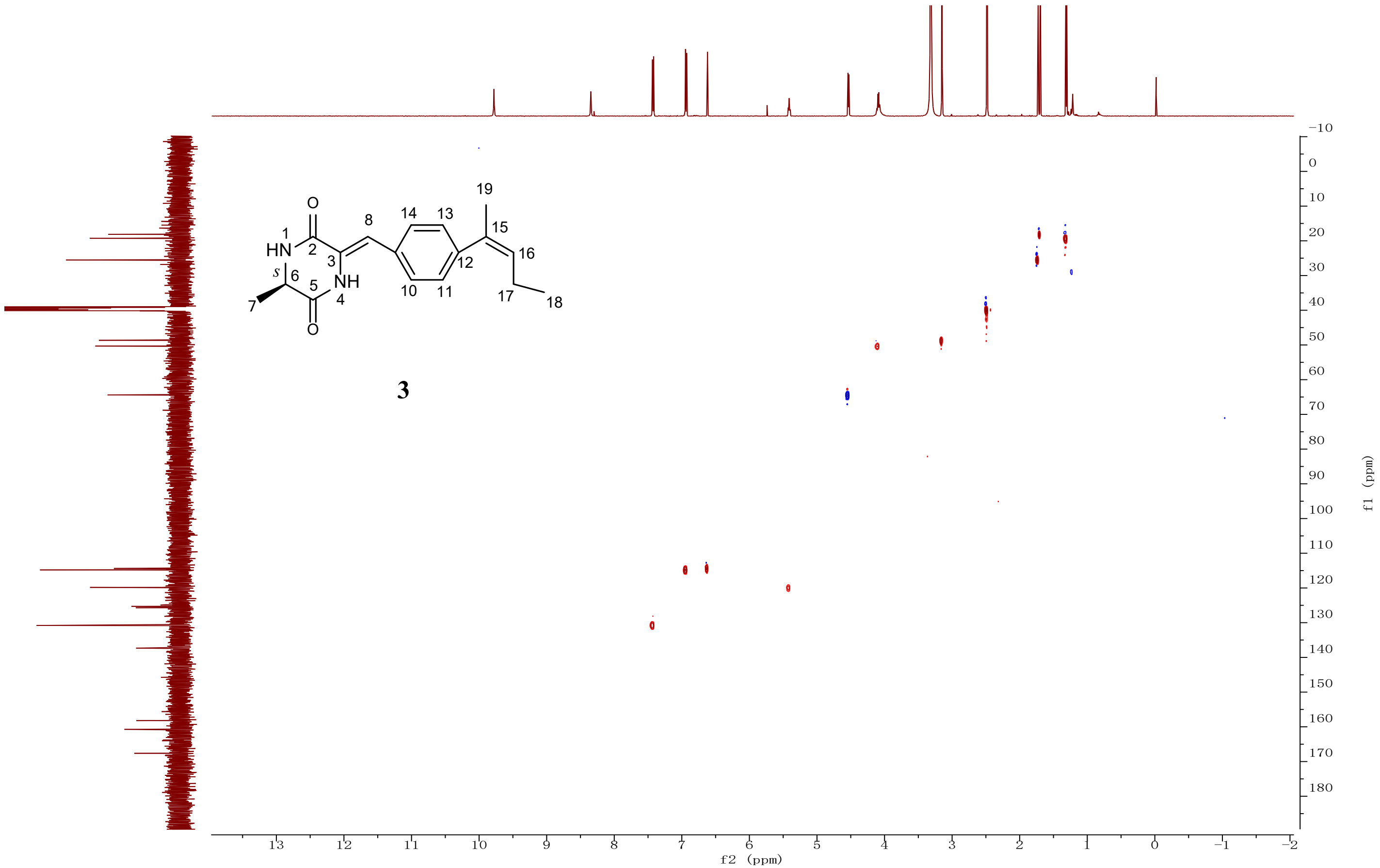

Figure S19. HSQC spectrum of compound **3** in DMSO-d_6.

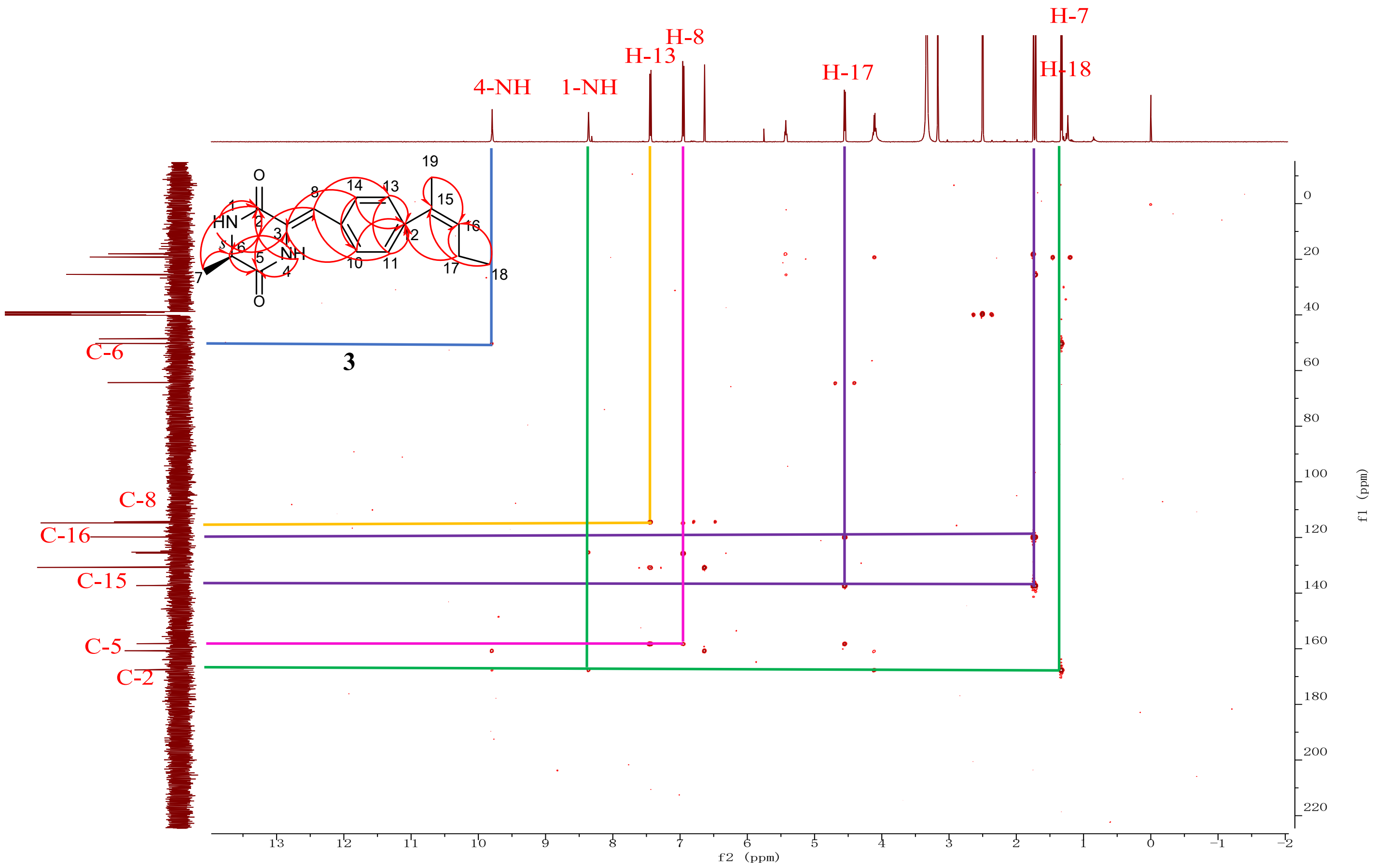

Figure S20. HMBC spectrum of compound **3** in DMSO-d_6.

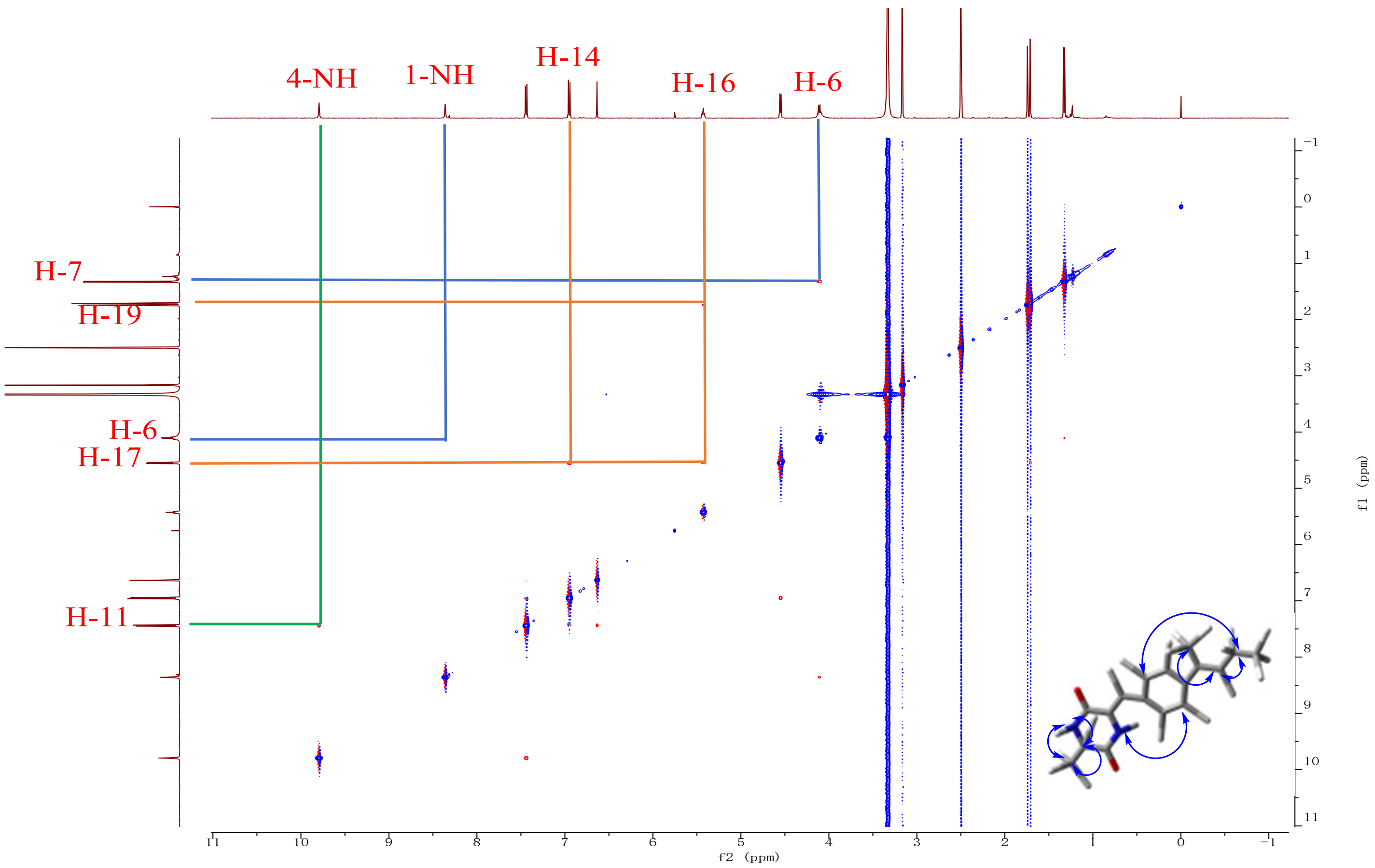

Figure S21. NOESY spectrum of compound **3** in DMSO-d_6.

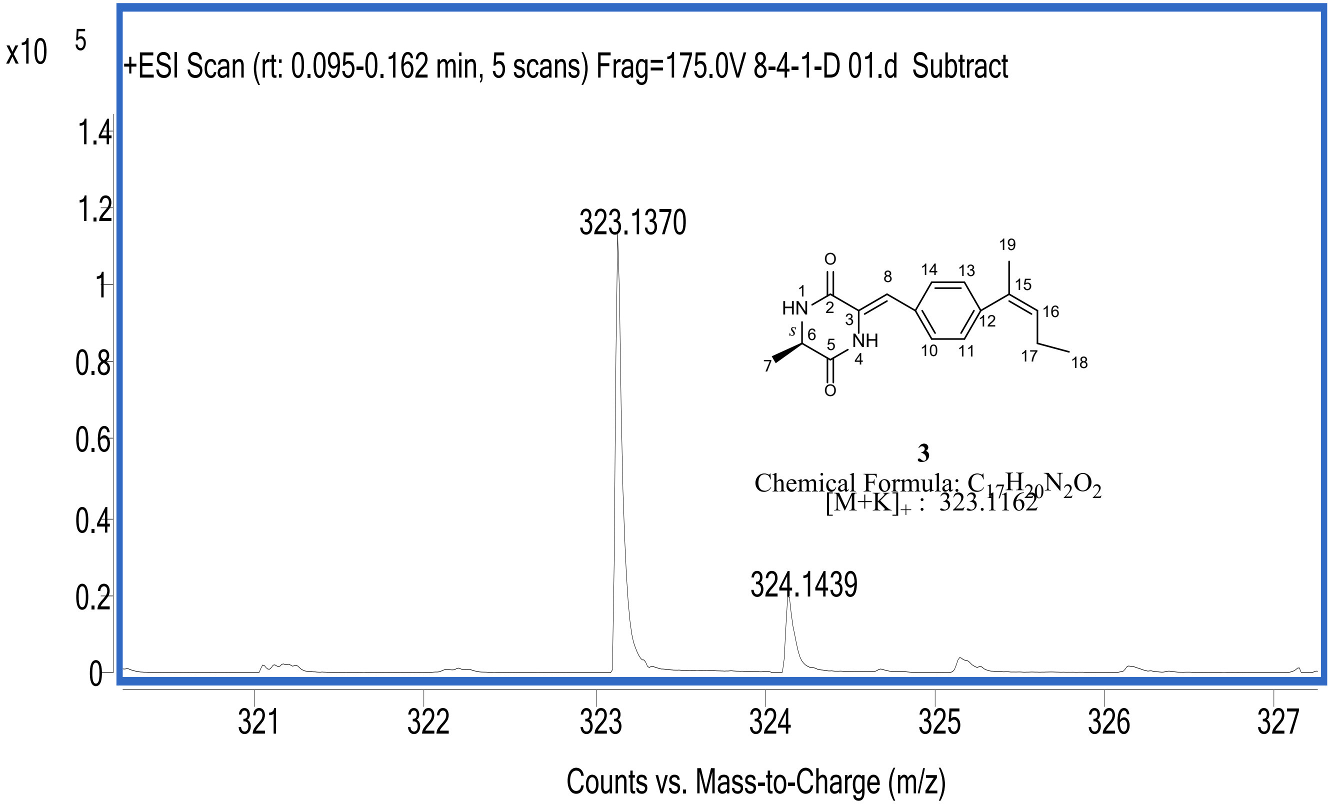

Figure S22. HRESIMS spectrum (positive ion mode) of compound **3**.

Table S1. The activity results about antitumor of compound **3**.

Compound	Concentration(μM)	Cell inhibition±SD（%）			
		K-562	786-O	TE-1	5637
3	20	12.54±0.90	10.50±2.44	3.12±2.51	-8.57±2.14
Compound	Concentration(μM)	Cell inhibition±SD（%）			
		A549	MKN-45	HCT 116	HeLa
3	20	0.34±2.89	-9.53±2.83	2.36±1.34	4.32±0.76
Compound	Concentration(μM)	Cell inhibition±SD（%）			
		GBC-SD	L-02	MCF7	HepG2
3	20	9.14±0.40	0.68±2.33	-2.20±3.23	-9.74±3.55
Compound	Concentration(μM)	Cell inhibition±SD（%）			
		SF126	DU145	CAL-62	PATU8988T
3	20	-0.89±3.61	-1.23±3.79	-4.54±3.43	-13.91±1.81
Compound	Concentration(μM)	Cell inhibition±SD（%）			
		HOS	A-375	A-673	293T
3	20	2.55±4.62	-11.95±0.26	8.17±2.02	0.14±1.80

Table S2. The activity results about anti-inflammatory of compounds **1−3**.

	**1**	**2**	**3**	dexamethasone
Inhibition±SD（%）	26.31±11.58	28.92±3.49	21.08±9.24	25.87±15.16
